# Phage Display as a Promising Platform for Peptide Drug Discovery

**DOI:** 10.3390/ph19081265

**Published:** 2026-08-11

**Authors:** Babak Bakhshinejad, Andreas Kajer

**Affiliations:** 1 Cluster for Molecular Imaging, Department of Biomedical Sciences, University of Copenhagen, DK-2200 Copenhagen, Denmark; babak.bakhshinejad@sund.ku.dk; 2 Department of Clinical Physiology and Nuclear Medicine, Copenhagen University Hospital-Rigshospitalet, DK-2100 Copenhagen, Denmark

**Keywords:** biopanning, combinatorial libraries, drug discovery, peptide therapeutics, phage display

## Abstract

Peptides have attracted increasing popularity in modern drug discovery, with the potential to address unmet clinical needs that lie beyond the reach of small molecules and antibodies. By offering “best-of-both-worlds” features, peptides are emerging as powerful tools for targeting disease-associated, challenging proteins. Combinatorial peptide libraries have played a crucial role in the development of peptide drugs. Phage display is one of the most widely adopted combinatorial frameworks for pharmaceutical exploration, ushering in a new paradigm in peptide drug discovery over the past few decades. Phage display libraries provide a vast repertoire of ligands that can be searched through biopanning to identify peptides with high specificity and affinity for a diverse range of biologically relevant targets. Although biopanning has demonstrated great potential in identifying target-specific ligands, many peptides identified by phage display still suffer from poor pharmacological properties, creating hurdles in translating initial hits from phage display selections into therapeutic molecules. To achieve improved pharmacological characteristics, various modifications can be introduced into peptides, such as cyclization, the incorporation of non-canonical amino acids, the introduction of D-amino acids, oligomerization, conjugation/fusion, and stapling. Optimizing peptide properties through these strategies has laid a solid groundwork for a significant number of phage display-derived peptides to progress from fundamental research and preclinical studies into various phases of clinical evaluations, with several peptide drugs already receiving regulatory approval for routine clinical applications. These achievements raise hopes for peptide phage display to remain a promising platform for drug discovery in the years ahead.

## 1. Introduction

Drug discovery is a complex, costly, and time-consuming process that involves the identification, design, and development of new compounds with therapeutic properties. On average, it takes more than a decade for a drug to cover the path from initial discovery to final approval [1]. Prior to the twentieth century, most drug discoveries relied on trial and error or, not uncommonly, serendipity. However, remarkable breakthroughs in different scientific disciplines led drug discovery to evolve into a more rational process. The advent of advanced techniques for determining the molecular structure of drugs, molecular modeling, state-of-the-art molecular biology methods, combinatorial chemistry, large biological libraries of biomolecules, high-throughput screening (HTS), and omics technologies made fundamental contributions to the establishment of the new era of drug discovery in the twentieth century [2]. These breakthroughs have enabled rapid preparation and assessment of a substantial number of biologically active molecules, as well as shortened the time needed for drug screening or selection endeavors and hit-to-lead development. In addition to expediting drug discovery, these technologies have resulted in a more profound understanding of the molecular mechanisms of diseases. From the very beginning of modern drug discovery, peptides have significantly contributed to the development of therapeutic compounds. This pivotal status results from particular characteristics of these molecules, representing them as a well-established class of therapeutics with the potential to address unmet medical needs that lie beyond the reach of conventional entities. Combinatorial peptide libraries have played an indispensable role in contemporary drug discovery efforts [3]. Phage display is one of the most important and widely used combinatorial peptide platforms that have revolutionized drug discovery by enabling the identification of peptides with high specificity and affinity for a wide variety of biological targets and offering a powerful means for the development of precision therapies [4]. The aim of the current review is to provide readers with clear insights into the role of phage display technology in the discovery and development of peptide drugs, with a particular focus on modification strategies used to optimize the pharmacological properties of peptides for moving them from fundamental research to clinical translation. Also, peptide drugs developed by phage display that have received FDA approval and are routinely used in the clinic will be reviewed.

## 2. Peptides and Drug Discovery: Expanding the Druggable Space Beyond Small Molecules and Large Antibodies

Developing small molecules that can bind with high affinity and specificity to clinically relevant target proteins, such as disease-associated enzymes and receptors, is considered a significant challenge for both academic and industrial research [5]. Despite decades of growing investment in drug research and development (R&D), the pharmaceutical industry is encountering stagnation in the number of new FDA-approved drugs. The depletion of low-hanging fruits in drug discovery has widened the cleft between cost and benefit, highlighting an immediate demand to delve into and discover new drug classes beyond small molecules. Biologics have shown promise to meet the rising demand for new therapeutic compounds [6]. For many decades, peptides, as an important class of biologics, have been an invaluable source of inspiration for drug development. For instance, penicillins, which are enzymatically modified tripeptides, are one of the most broadly used categories of peptide-based therapeutics [7]. Peptides play essential roles as biologically active molecules in the form of growth factors, antigens, cytokines, neurotransmitters, ion channel ligands, anti-infective agents, and hormones [8,9,10]. The first medical application of peptides as a drug dates back to the early decades of the 20th century, when insulin was extracted from the pancreas of dogs and successfully utilized to treat type 1 diabetes [11,12]. Since then, peptides have been extensively used in medicine, establishing a unique and prominent position in the pharmaceutical arena.

Due to some unique pharmaceutical characteristics, peptides have attracted considerable attention in modern drug discovery [13]. Peptides represent a distinct drug modality, occupying the chemical space between small molecules and larger biologics. By combining the favorable bioavailability of small molecules and the target-binding specificity of antibodies, they offer “best-of-both-worlds” features, making them promising candidates for targeting challenging proteins [14]. Over the recent decades, an increasing interest has been focused on pharmacologically relevant targets that are not effectively druggable by small molecules and antibodies because of their shallow or extended binding surfaces. Peptides, however, can interact with hydrophobic pockets typically targeted by small molecules, providing interesting probes for identifying druggable sites on proteins [15]. Furthermore, they rival antibodies in their ability to selectively and potently modulate targets with extended binding interfaces. They can bind to cell surface receptors with high affinity and specificity and induce intracellular signaling effects, comparable to antibodies [16]. Beyond their therapeutic properties, peptides are emerging to play a growing role as powerful tools for target discovery and validation. Peptides provide some advantages similar to those of small molecules, such as efficient tissue and organ penetration, while offering target specificity superior to small molecules. In addition, their reduced immunogenicity, more convenient and less costly production in large quantities, and greater surface density (more peptide molecules per surface unit) with resultant higher activity per mass are considered merits over antibodies [17,18]. Some properties of peptides, like target selectivity, fast clearance by the kidney, biological half-life, and amenability for modifications, make them especially helpful for molecular imaging (as imaging probes) and drug delivery (as carriers of toxic payloads in peptide-drug conjugates) [17]. Table 1 provides a comparison of the various properties of peptides, small molecules, and antibodies.

The cutting-edge advancements in the field of peptide therapeutics have led to the development of approximately 100 peptide drugs for various diseases currently available in the global market. In addition, hundreds of peptides are undergoing preclinical evaluations or different stages of clinical development [19]. Peptide drugs represent a significant proportion of the global pharmaceutical market. The global market for peptide pharmaceuticals has been expanding at a compound annual growth rate (CAGR) of 10.2%, with the growth of worldwide sales from 41.44 billion USD in 2023 to 45.66 billion USD in 2024, highlighting a significant increase in market value. The market of therapeutic peptides is poised for substantial expansion in the coming years, with its value forecasted to reach 68.83 billion USD by 2028, driven by a CAGR of 10.8%. In 2023, North America was the largest region in the therapeutic peptide market, while the Asia-Pacific region is expected to exhibit the highest growth rate in the years ahead (throughout the forecast period). A key driver for this market expansion is the increasing prevalence of chronic diseases, which continues to boost the demand for peptide therapies [20]. Despite the above-mentioned outstanding benefits, peptides suffer from some drawbacks, such as sensitivity to proteolytic degradation and poor in vivo stability. Nevertheless, it is possible to address these limitations by introducing a variety of modifications into peptides to optimize their biochemical and biophysical properties, including affinity, charge, hydrophobicity, solubility, and stability for preclinical and clinical applications. The diverse strategies used for peptide modification are discussed extensively in Section 5.

## 3. Phage Display and Advancing Drug Discovery: Searching in Vast Combinatorial Peptide Libraries for Finding Therapeutics

Historically, peptide drug discovery was primarily dependent on serendipity or the isolation of active components from natural products. With the rise of combinatorial chemistry [21] as well as the reverse pharmacology approach [22], which is based on identifying a particular disease-related target followed by large-scale screening of chemical libraries, these laborious trial-and-error processes were supplanted by high-throughput library screening to discover novel peptide leads for therapeutically relevant targets [23]. The contemporary revolution in molecular biology and the advent of recombinant technology led to the introduction of biological display systems by which genetically encoded combinatorial libraries, orders of magnitude larger than chemical libraries, are generated and undergo evolutionary selection to recognize candidate leads suitable for drug discovery. By mimicking natural evolution, biological libraries provide an opportunity to search in highly diverse libraries for finding candidates with strong target-binding affinities. Display technologies play an essential role in modern peptide drug discovery, and among them, phage display is the most extensively adopted platform for discovering promising therapeutic peptides. This powerful display approach for peptide discovery against biological targets made its debut in the mid-1980s by George P. Smith, who earned the Nobel Prize in Chemistry in 2018 for his pioneering work on peptide phage display [24]. He shared this prize with Gregory Winter (for his work on antibody phage display) and Frances Arnold (for her work on directed evolution of enzymes) [25,26]. In his original article, Smith indicated that by cloning peptide-encoding sequences into the genome of bacterium-specific viruses, called bacteriophages or phages, peptides could be displayed on the exterior of the phage particle as a fusion to one of its coat proteins [27]. The concept of combinatorial peptide libraries has been inspired by nature. Arachnids (spiders and scorpions) and cone snails are natural combinatorial peptide chemists that have developed genetic mechanisms for generating optimized combinatorial peptide libraries in their venoms. It has been estimated that tens of thousands of toxic peptides are encoded by the genome of these organisms. These venom peptides serve as a rich source of bioactive molecules with unique disulfide-constrained structures that act as neurotoxic, antimicrobial, and cytolytic agents and play key roles in prey capture, defense, and competitor deterrence [28].

**Figure 1:**
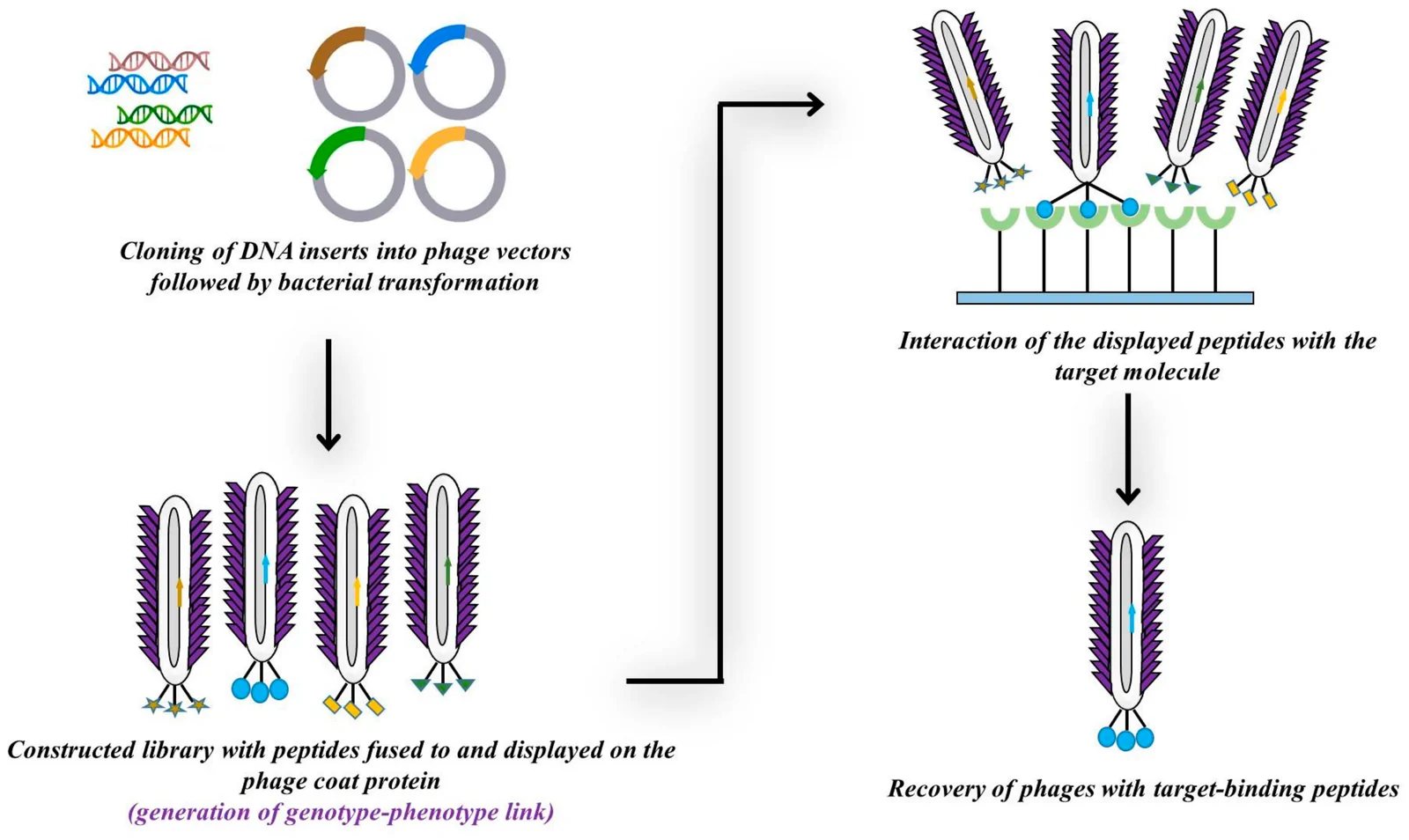
Overview of the peptide phage display workflow. DNA fragments encoding diverse peptide sequences are cloned into phage vectors, which are subsequently introduced into bacterial host cells by transformation to construct a combinatorial phage display library. The encoded peptides are expressed as fusions to the minor coat protein (pIII) of M13 phage and displayed on the phage surface, thereby establishing a physical link between the displayed peptide (phenotype) and its encoding DNA (genotype). The resulting phage library is then exposed to the target molecule, allowing phage particles displaying target-specific peptides to bind to the target, leading to their selective recovery for subsequent affinity selection and sequence identification.

Building upon the foundational principles of combinatorial chemistry and genetic diversity, phage display takes advantage of the potential of recombinant technology to create molecular diversity that represents an astounding number of unique peptides displayed on the surface of more commonly non-lytic filamentous or less commonly lytic head and tail phages. This vast repertoire of peptides is a worthwhile treasure for many high-affinity binders with therapeutic potential [29,30]. The huge diversity of the library is a significant contributory factor in a successful phage display selection. In addition to the ability to construct large libraries, another fundamental feature of phage display as a powerful discovery modality is the physical linkage between genotype (the genetic material that encodes the displayed peptide) and phenotype (the displayed peptide), which allows for determining the amino acid sequences of peptides displayed on isolated phage clones [31]. Figure 1 schematically illustrates the genotype-phenotype link underlying phage display and provides a general overview of the peptide phage display workflow. Phage display peptide libraries have been used for a variety of biomedical purposes, such as epitope mapping [32], selection of agonists or antagonists for pharmacologically significant receptors [33,34], identification of cell-targeting peptides [35], targeted delivery of drugs and therapeutic genes [36,37], molecular imaging [38], and proteomics (analysis of protein–protein interactions) [39].

To place phage display in the broader landscape of peptide discovery technologies, it is useful to briefly compare it with other combinatorial library platforms. This comparison with alternative approaches highlights the unique features that have made phage display so broadly applicable. These alternatives include synthetic chemical libraries and biological display systems [40]. While synthetic chemical libraries offer broad chemical diversity and enable the incorporation of unnatural amino acids, they generally lack the direct genotype–phenotype link that facilitates rapid identification of selected ligands and are typically limited in library size. Biological display technologies overcome this limitation through the physical connection of the displayed peptide to its encoding genetic information. Among these, cell-based platforms, such as bacterial and yeast display, provide efficient protein expression and folding and are compatible with fluorescence-activated cell sorting (FACS), although their library diversity is constrained by transformation efficiency [41,42]. In contrast, cell-free systems, such as ribosome and mRNA display, enable the construction of much larger libraries but are technically more demanding and less suitable for selections against complex biological targets [41]. Phage display occupies a unique position at the interface between cellular and acellular display technologies. Similar to cell-based systems, phage libraries are generated and propagated in living bacterial cells, providing a simple, inexpensive, and highly efficient means of constructing, amplifying, and reusing large combinatorial libraries with standard laboratory equipment. However, affinity selection is performed independently of the cellular context, allowing phage-displayed peptides to be screened against an exceptionally broad range of targets, including purified biomolecules, cells, tissues, and even living animals or human patients [43]. In addition, phage particles are remarkably robust and tolerate diverse selection conditions, including stringent washing procedures involving high salt concentrations, low pH, and denaturing agents such as urea. By combining the practical advantages of both cellular and acellular approaches with the unique genotype–phenotype link that enables straightforward identification of selected ligands, phage display has become one of the most versatile, accessible, and widely adopted platforms for peptide discovery and the successful translation of peptide ligands into therapeutic applications.

Despite its considerable strengths, phage display is not without intrinsic limitations that can influence the reliability of downstream selection outcomes. In phage display, peptides are often fused to pIII, a minor coat protein that mediates binding to the F pilus of the bacterial host and is essential for M13 phage infection and assembly [44]. However, the insertion of exogenous peptides can impose structural constraints on the membrane translocation of the pIII fusion protein and its subsequent folding in the periplasm of the host bacterium. For some peptide sequences, these constraints are sufficiently severe that the phage cannot tolerate their display, resulting in little or no presentation of the corresponding peptides on the phage surface. Consequently, such sequences are effectively censored from the library because they compromise pIII function, thereby reducing phage infectivity and the efficiency of phage assembly [45]. This sequence censorship can eliminate potentially valuable peptide candidates before selection, ultimately reducing the effective diversity of the library. Although sequence censorship reduces the diversity of the input library, an even greater challenge arises during the affinity selection process itself. The major bottleneck in biopanning is the frequent isolation of nonspecific binders—phage clones that are recovered despite lacking genuine affinity for the intended target. These can be broadly classified into two categories: selection-related and propagation-related nonspecific binders [46]. Selection-related nonspecific binders arise from unintended interactions between either the phage body or the displayed peptide and components of the selection system, such as the polystyrene surface of the solid support. Indeed, numerous polystyrene-binding peptides have been identified in phage display studies [47]. Hydrophobic amino acids, particularly the aromatic residues phenylalanine, tyrosine, and tryptophan, play key roles in mediating interactions with polystyrene surfaces [48,49]. In addition, the large body of M13 phage can impose steric constraints that limit access of the displayed peptide to the target. Furthermore, the close proximity of neighboring coat proteins and displayed peptides may further hinder productive binding [50], thereby contributing to the recovery of selection-related nonspecific binders. These interactions introduce background noise that is independent of true target recognition and can complicate or mislead the identification of specific binders. In contrast, propagation-related nonspecific binders originate from differences in the growth behavior of individual phage clones in bacterial hosts. In these cases, certain clones amplify more efficiently, and this propagation advantage can lead to their enrichment regardless of binding affinity. Mechanistically, such advantages stem from alterations in the phage genetic material [46]. Recently, specific sequence motifs within the displayed peptide have also been suggested to be associated with enhanced amplification rates [51]. Overall, clones with superior propagation properties can dominate the recovered pool, while authentic binders with slower amplification rates are progressively lost. Consequently, enrichment during biopanning does not necessarily reflect true binding affinity. This decoupling highlights the importance of careful experimental design and the use of complementary validation strategies to reliably distinguish genuine target-specific binders from selection- and amplification-related artifacts.

Various bacteriophages have been exploited as platforms for peptide display, with filamentous phages and lytic tailed phages being the two principal classes. Each display system possesses unique biological characteristics that confer distinct advantages and limitations, making their suitability highly dependent on the intended application. The fundamental distinction between these two groups lies in their life cycle. Filamentous phages, such as M13, assemble in the bacterial periplasm and are continuously extruded through the cell envelope without lysing the host cell. In contrast, lytic phages, such as T7, assemble entirely within the bacterial cytoplasm and are released only after host cell lysis [52]. Consequently, peptides displayed on lytic phages do not need to traverse the bacterial secretion machinery or the cell membrane during phage assembly. However, the membrane-associated assembly pathway of filamentous phages imposes important constraints on the molecules that can be displayed. Coat proteins must first be inserted into the bacterial membrane before phage assembly, and peptide fusions that interfere with membrane translocation, phage assembly, or particle extrusion are often poorly tolerated [45]. Likewise, highly hydrophobic sequences, peptides with complex conformations, or large inserts may impair coat protein function, thereby reducing phage infectivity or propagation efficiency. These biological constraints can introduce biases during library construction and selection. Lytic phages largely circumvent these limitations because phage assembly occurs in the cytoplasm, eliminating the requirement for secretion through the bacterial envelope [42]. As a result, they are generally more tolerant of large fragments and structurally complex biomolecules, owing both to their intracellular assembly pathway and to the greater packaging capacity of their genomes for exogenous DNA. Furthermore, displayed peptides are not constrained by compatibility with the host secretion or infection machinery. Consistent with these features, peptide libraries displayed on T7 phage have been reported to exhibit greater diversity and reduced sequence bias compared with M13-derived libraries [53]. Despite these advantages, lytic phage display systems also present several practical limitations. Their relatively large genomic vectors are generally more cumbersome to manipulate genetically than filamentous phages, and because assembly occurs in the reducing environment of the bacterial cytoplasm, proteins requiring disulfide bond formation may not fold correctly, restricting the display of certain classes of peptides [52,54]. In contrast, filamentous phages possess several attributes that have established them as the most widely used platforms for phage display. Their genomes are readily manipulated, tolerate insertions within non-essential regions, and can be isolated in both single-stranded and double-stranded forms, greatly facilitating cloning and library construction. Unlike lytic phages, the length of filamentous virions can increase to accommodate larger genomes without compromising particle assembly [52,55]. Moreover, their non-lytic mode of propagation enables continuous production and accumulation of high phage titers from viable bacterial cultures [55], simplifying amplification and library handling. Together, their remarkable genetic flexibility, ease of manipulation, efficient propagation, and the extensive molecular toolbox developed over several decades have made filamentous phages—particularly M13—the workhorse of phage display technology. Overall, each phage display platform offers distinct strengths and limitations, and the optimal choice depends on the biological characteristics of the target and the objectives of the selection strategy. Although numerous phage display systems have been developed, M13 filamentous phage remains the preferred and most extensively used platform owing to its versatility, robustness, and well-established methodology. Nevertheless, rather than being viewed as competing technologies, filamentous and lytic phage display systems should be regarded as complementary platforms, each expanding the range of molecules and applications accessible through phage display.

## 4. Biopanning and Affinity-Driven Peptide Discovery: Unveiling Target-Specific Binders from Phage Display Libraries

**Figure 2:**
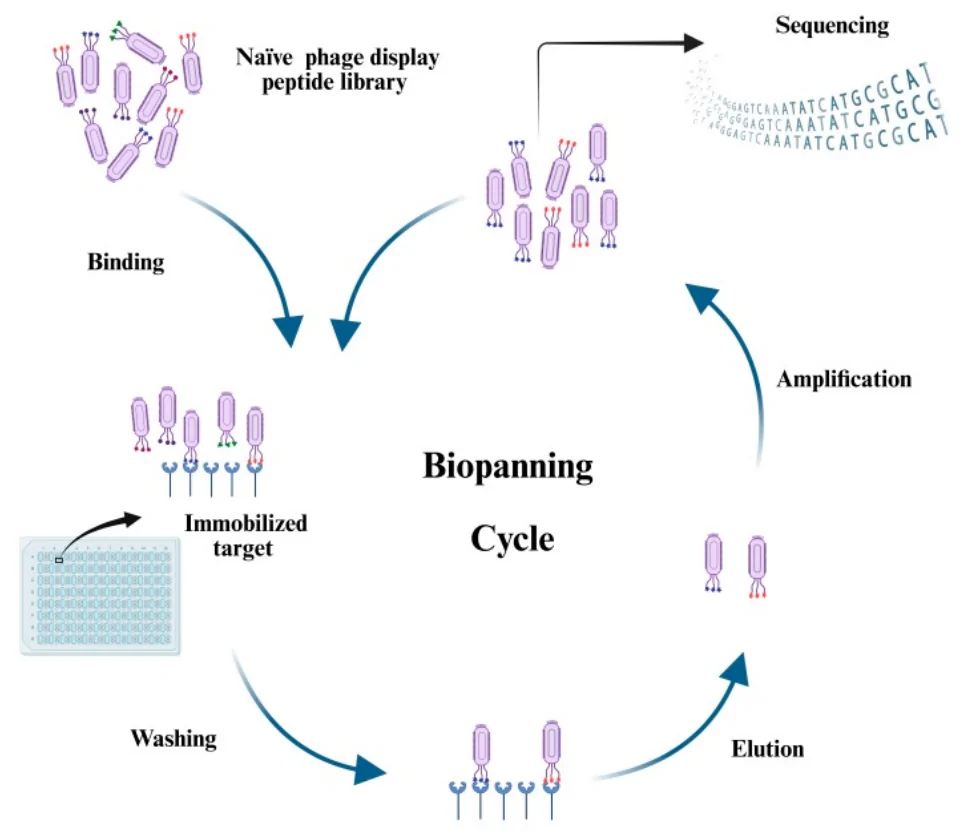
A schematic illustration of biopanning. The affinity selection of phage display peptide libraries through biopanning begins with presenting the pool of virions in the library, displaying a diverse range of unique peptides, to the target immobilized on a solid phase (e.g., microtiter plate). After stringent washing, the target-bound phages are recovered by elution, and the eluted phages are amplified by infecting the host bacterial cells. This cycle might be repeated for several rounds. At the end of biopanning, a part of the genome in recovered and amplified phages that encodes the displayed peptide is sequenced to identify the amino acid sequences of peptides displayed on the relevant isolated clones.

To find target-specific peptides, the phage display library needs to undergo affinity selection through a typically cyclic procedure that is called panning or biopanning [56]. The different steps of the biopanning cycle are indicated in Figure 2. Biopanning commences with presenting the library to the target of interest. The target-exposed library is subjected to a series of stringent washes to discard unbound or weakly bound phages, and the bound phages are then eluted. Afterward, the recovered phage pool is amplified by infecting the host bacterium [57,58]. The primary objectives of selection and amplification are (1) to isolate phages whose displayed peptides bind specifically to the target and (2) to enrich these isolated phage clones, respectively. Therefore, library members with high fitness (phage clones displaying peptides with high binding affinity for the target) are expected to survive and reproduce among a diversified and large repository of phages, passing on their genotype (and the corresponding phenotype) to the next generation. In an optimal condition, a single round of biopanning can enrich target-binding clones by several orders of magnitude. In conventional biopanning, the steps of selection and amplification are repeated for several rounds to achieve a greater enrichment factor of target-binding peptides. After the final cycle, the primary structure of peptides displayed on retrieved phages is identified by sequencing a part of the phage DNA harboring the peptide-encoding nucleotides through the Sanger approach. This low-throughput sequencing (LTS) strategy relies on subjecting a limited number (typically tens) of clones to peptide sequence identification. A critical consideration in biopanning design is that recombinant target proteins are commonly immobilized onto a solid support for affinity selection. However, immobilization can induce conformational changes or partial denaturation of the target protein [59], potentially occluding or distorting native binding sites. As a result, some peptide–target interactions may be hindered because relevant epitopes become inaccessible to members of the phage display library. Beyond target presentation, another important consideration is the choice of elution strategy, which can profoundly influence the outcome of affinity selection but is often overlooked. Bound phages are most commonly recovered by nonspecific elution with an acidic buffer [57]. Acidic elution acts as a general binding disruptor because the low pH can induce conformational changes in the target protein and weaken multiple types of molecular interactions, resulting in the release of virtually all bound phages regardless of their binding epitope or functional relevance. When a high-affinity ligand for the target is available, however, competitive elution is often the preferred strategy. Ideally, a highly specific ligand that binds to the target with high affinity is used to displace phages recognizing the same or overlapping binding site. Alternatively, when feasible, the free target molecule itself can serve as a competitive eluent [55,57,60,61]. The key advantage of competitive elution over nonspecific elution methods is that it introduces biological selectivity into the elution step, rather than relying purely on bond disruption. Unlike acidic elution, which indiscriminately releases most bound phages by disrupting protein–ligand interactions, competitive elution preferentially enriches phages displaying peptides that recognize the same or an overlapping binding site as the competing ligand. Although some nonspecifically bound phages or phages recognizing unrelated epitopes may also be recovered owing to incomplete washing or mechanical dissociation, competitive elution generally increases the proportion of functionally relevant binders in the eluted pool. As a result, subsequent selection rounds are more likely to favor peptides targeting the desired functional epitope. However, this increased selectivity comes at the expense of reduced epitope diversity, as peptides recognizing alternative binding sites may remain unrecovered and consequently be underrepresented or lost using competitive elution. The utility of biopanning has been demonstrated in numerous peptide discovery studies spanning diverse biomedical fields. High-affinity peptides have been isolated against cancer-associated targets such as EGFR [62], HER2 [63], FGFR [64], PSA [65], VEGF [66] and CD44 [67], where they have been explored for tumor imaging, targeted drug delivery and precision therapeutics. Likewise, biopanning has yielded peptide ligands recognizing viral and bacterial antigens for diagnostic and antimicrobial applications [68,69,70,71,72,73,74], as well as peptides targeting molecules involved in inflammation and immune processes with potential therapeutic value [75,76,77,78,79]. Collectively, these representative studies demonstrate that biopanning is not only an efficient affinity selection strategy but also a versatile platform for discovering functional peptide ligands with broad biomedical and translational applications. In recent years, phage display has been coupled with a high-throughput sequencing strategy called next-generation sequencing (NGS), which enables the parallel sequencing of a large number (thousands to millions) of isolated clones [80,81]. NGS provides a deeper insight into peptide enrichment, selection dynamics, and sequence diversity of phage pools, empowering a quantitative and data-driven selection of peptides from phage display libraries. Before biopanning, NGS can be applied to naïve phage display libraries as a powerful quality control tool, enabling comprehensive characterization of their sequence composition and identification of amino acid biases [81,82]. Detecting such compositional biases before selection provides valuable information about the initial diversity and representation of peptide variants within the naïve library, allowing researchers to make more informed decisions regarding selection strategies and the interpretation of downstream biopanning results. In addition to library quality assessment, NGS allows researchers to monitor changes in sequence abundance across successive rounds of selection. Such population-level analyses have redefined biopanning as a quantitative evolutionary experiment, in which the trajectories of millions of peptide variants can be tracked simultaneously throughout the selection process. Because NGS offers a population-wide view of the selection output, promising peptide candidates can be identified after fewer rounds of biopanning, thereby reducing the overall number of selection cycles required. Consistent with this, it has been found that even one round of NGS-based phage display can be used to identify target-binding peptides [83]. This non-cyclic workflow of phage display selection can also significantly diminish the bias linked to amplification that occurs between rounds of selections [45]. Amplification-associated bias is capable of misdirecting the identification of promising binders, posing a great challenge to the genuineness and integrity of phage display selections [45,47]. NGS-assisted biopanning has been successfully applied to identify peptide ligands against a variety of clinically relevant targets [84,85,86,87,88].

The conventional biopanning workflow can be tailored to reduce the likelihood of retaining and isolating nonspecific background binders by incorporating a negative selection step. In negative selection, the input phage library is first incubated with all components of the selection system except the target of interest. The primary objective is to deplete the library of phage clones that bind to the selection matrix, blocking agents, carrier proteins, or other experimental components, thereby reducing the enrichment of selection-related nonspecific binders. The phage population recovered from this negative selection step is subsequently used as the input for positive selection against the intended target [89]. The terms negative biopanning and subtractive biopanning are frequently used interchangeably in the phage display literature because both refer to the removal of undesirable background-binding phage clones. However, a more precise distinction can be made based on the nature of the target used during the depletion step. Whereas negative biopanning generally refers to eliminating phages that recognize non-target components of the selection system, subtractive biopanning is more commonly applied when the target of interest is embedded within a complex biological system, such as whole cells, tissues, or organisms. In these cases, the library is first exposed to a closely related but undesired biological target—for example, healthy cells before diseased cells, or one cell type before another—to remove phages that bind to shared molecular features. The remaining phage population is subsequently subjected to positive panning against the desired target, thereby increasing the likelihood of isolating peptides that recognize molecular features unique to the complex biological target [35]. Despite its advantages, negative selection is not without limitations and cannot be expected to achieve complete and selective depletion of undesirable phage clones. Ideally, the purpose of this modification in the biopanning protocol is to eliminate phages whose displayed peptides interact with non-target components of the selection system. In practice, however, nonspecific interactions may also arise from the phage particle itself rather than from the displayed peptide. Given that the filamentous phage is orders of magnitude larger than the displayed peptide and presents a substantial proteinaceous surface, it can interact with matrices, blocking agents, carrier proteins, or other components of the selection system independently of the peptide insert [50]. Consequently, phages displaying genuine target-binding peptides may nevertheless be removed during the depletion step because of nonspecific interactions mediated by the phage body. Negative selection therefore represents a trade-off between improving selection specificity and preserving library diversity. While it effectively reduces the enrichment of background binders, it may also unintentionally eliminate phage clones displaying biologically relevant peptides, thereby decreasing the probability of recovering optimal target-specific binders in subsequent rounds of selection.

One of the major innovations in phage display technology is in vivo biopanning, which enables affinity selection to be performed directly in living animals rather than against isolated targets under artificial in vitro conditions. Unlike many other combinatorial peptide library technologies that rely predominantly on in vitro workflow for peptide isolation, phage display uniquely permits the physical link between peptide phenotype and encoding genotype to be maintained throughout systemic circulation and tissue recovery, making in vivo selection feasible. In a typical in vivo biopanning experiment, a phage display library is administered into the circulation of an experimental animal, allowed to distribute throughout the body, and phages that preferentially home to a target tissue or organ are subsequently recovered and amplified [90]. By preserving the native physiological environment—including vascular architecture, extracellular matrix, blood flow, cellular heterogeneity, and receptor accessibility—in vivo biopanning enables the identification of tissue- and organ-homing peptides that are often difficult or impossible to isolate using conventional in vitro approaches. Since its introduction, in vivo biopanning has emerged as a powerful strategy for peptide discovery, and numerous studies have demonstrated its ability to identify physiologically relevant tissue-homing and target-selective peptides with considerable diagnostic and therapeutic potential [90,91,92,93,94,95,96]. More recently, the integration of in vivo biopanning with NGS has further enhanced the power of peptide discovery by combining physiological selection with comprehensive population-level sequence analysis. This integrated platform enables the identification of enriched peptide ligands with greater accuracy and quantitative resolution, and its successful application in numerous studies highlights its remarkable potential for accelerating peptide discovery [97,98,99,100,101]. Another further methodological advancement in phage display selection has been its integration with microfluidic-based platforms, which can optimize various aspects of phage display technology by providing highly controlled selection environments throughout the biopanning workflow [102]. By enabling precise control over fluid flow and mass transport, microfluidic systems enhance interactions between phage particles and target molecules while allowing stringent and reproducible washing conditions. Unlike conventional biopanning, which relies on repeated centrifugation and manual washing steps that are labor-intensive and can result in the loss of valuable phage clones, microfluidic devices employ specifically designed microchannels and fluid dynamic or acoustic forces to efficiently remove unbound phages while retaining high-affinity binders [103]. Furthermore, droplet-based microfluidic platforms can encapsulate individual phages together with target-coated beads in water-in-oil droplets, enabling single-clone amplification and binding within isolated environments. Because amplification occurs in uniform monodisperse droplets, all phage clones are propagated under comparable conditions, minimizing amplification bias arising from differences in phage propagation rates [104,105]. By integrating key selection steps—including target presentation, washing, amplification, and phage recovery—into a miniaturized and automated platform, microfluidic technologies offer greater control over the biopanning process while reducing reagent consumption, shortening experimental time, and improving reproducibility [102]. Consequently, the merge of phage display with microfluidic technology represents a promising complement to the established peptide discovery workflows.

## 5. From Biopanning Hits to Therapeutic Agents: Modifications to Overcome the Pharmacological Limitations of Peptides Discovered by Phage Display

Although biopanning has shown great promise in identifying specific ligands with high affinity for a wide range of biologically relevant targets, phage display-derived peptides are generally not sufficient to serve as drugs themselves and still suffer from poor pharmacological properties, such as a typically short half-life in the body, high sensitivity to enzymatic degradation, and limited penetration through the biological membranes, all of which result in the low bioavailability of these molecules in the in vivo milieu [106,107]. These limitations create hurdles for scientists to convert hits gained from phage display selection to therapeutic molecules. In addition, many peptides obtained from phage display show affinities in the micromolar range, making them unsuitable or non-optimal for most clinical applications. Many peptides completely lose their affinities or exhibit low affinities when synthesized as free molecules, highlighting that preserving the affinity and functional activity of peptides outside the phage context is challenging. Peptides are presented in multiple copies on the phage scaffold, enabling them to bind to their target, in particular cell-surface targets, through multivalent interactions. However, synthesized peptides are in monomeric form, leading to the complete loss or reduced affinity of the peptide out of the phage framework [40]. Over the recent decades, the scientific community and pharmaceutical industry have devoted extensive endeavors to translate peptides identified by phage display from basic research to clinical applications. These efforts have been focused on introducing various modifications into peptides to achieve improved pharmacokinetic and pharmacodynamic characteristics for preclinical and clinical applications. The relatively weak interactions in short peptides, such as hydrogen bonds, van der Waals interactions, and intramolecular hydrophobic effects, are typically insufficient to effectively stabilize secondary structures. Thus, some modifications aim to mimic, stabilize, or construct ideal secondary structures, including helices, turns, hairpins, and extended conformations, to improve the physicochemical characteristics of peptides [108,109]. In this section, the most important modifications that are commonly used to improve the different properties of peptides to make them a fit for clinical translation are discussed. A summary of these modification strategies, together with their major advantages, limitations, and effects on peptide properties, is provided in Table 2.

### 5.1. Cyclization

Cyclization is a useful strategy for optimizing peptide properties that can improve their pharmacological features, including binding affinity, specificity, stability, and protease resistance [110]. Cyclic peptides have emerged as highly effective inhibitors of protein–protein interactions (PPIs), and their larger and more structurally defined binding surfaces enable more efficient optimization for potency and selectivity, addressing a key challenge in drug discovery in targeting large and relatively featureless binding interfaces of protein molecules [111]. Linear peptides are flexible and can adopt many conformations. However, this structural adaptability and variability may reduce their binding affinity for the target. Furthermore, linear peptides must meet a minimum length requirement to achieve structural stability. As a result, libraries of long peptides (>40 amino acids), although not exhaustive, have demonstrated greater success in isolating target-specific binders compared to shorter peptide libraries [112]. By preorganizing intramolecular interactions, the cyclization of linear peptides allows them to form and reinforce secondary structure elements, such as loops, turns, α-helices, and β-sheets, that would otherwise be inaccessible to many single linear peptide sequences [16]. This structural constraint stabilizes the bioactive conformation of peptides by diminishing flexibility, thereby reducing the entropic penalty upon receptor binding and ultimately enhancing potency, specificity, and selectivity [113,114]. When displayed on the phage, the constrained structure caused by cyclization restricts the number of potential conformers, which can reduce the effect of the phage environment on the structure and function of the peptide. Therefore, cyclic peptides selected by phage display are more likely to retain their binding affinity for the target when synthesized as free molecules and removed from the phage scaffold [112]. Cyclization to create cyclic peptide libraries is typically performed by flanking a random peptide sequence with two invariant cysteine residues that generate disulfide bridges. A considerable body of evidence from the literature provides support for the often higher affinity of cyclic peptides than linear ones identified via phage display selection on the same target.

Methods for generating cyclic peptide libraries are classified in two broad categories: those based on genetic encoding and those based on chemical synthesis of cyclic peptides [115]. The development of these methods for producing very large libraries of cyclic peptides has significantly expanded the scope of hits discovered by combinatorial technologies against targets previously deemed undruggable. Highly diverse libraries of genetically encoded cyclic peptides are efficiently provided by phage display [116,117], split-intein circular ligation of peptides and proteins (SICLOPPS) [118], and mRNA display [119]. These methods utilize the genetically encoded transcription and translation machinery to generate a pool of cyclic peptides that either remain covalently attached to or are co-localized with their corresponding nucleotide sequences. The link between genotype and phenotype in these systems enables the convenient and rapid identification of peptide hits with desired binding or functional activity. In genetically encoded libraries, the random repertoire of amino acids serves as the source of diversity. Genetic encoding methods, in particular phage display, can produce large libraries with a huge diversity of unique cyclic peptides, are cost-effective for large screens, permit easy and straightforward identification of the most promising hits, and have proven useful in the efficient identification of peptides against a variety of biologically relevant targets. However, the chemical instability of disulfide bonds in biological systems and the limited capacity of conventional cyclization strategies for tuning the size and structural diversity of rings have led to the development of cyclic libraries based on chemical synthesis. Chemically synthesized cyclic peptides can be attained by approaches such as DNA-encoded chemical libraries (DECLs) [120,121] and one-bead-one-compound (OBOC) libraries [122,123], which have demonstrated remarkable success in identifying cyclic peptides, including PPI inhibitors. Each of the methods using genetic encoding or chemical synthesis of cyclic peptide libraries comes with its own pros and cons. To obtain further information about the potential of these approaches for generating cyclic libraries and identifying target-specific cyclic peptides, readers can refer to the references noted in the current paragraph.

**Figure 3:**
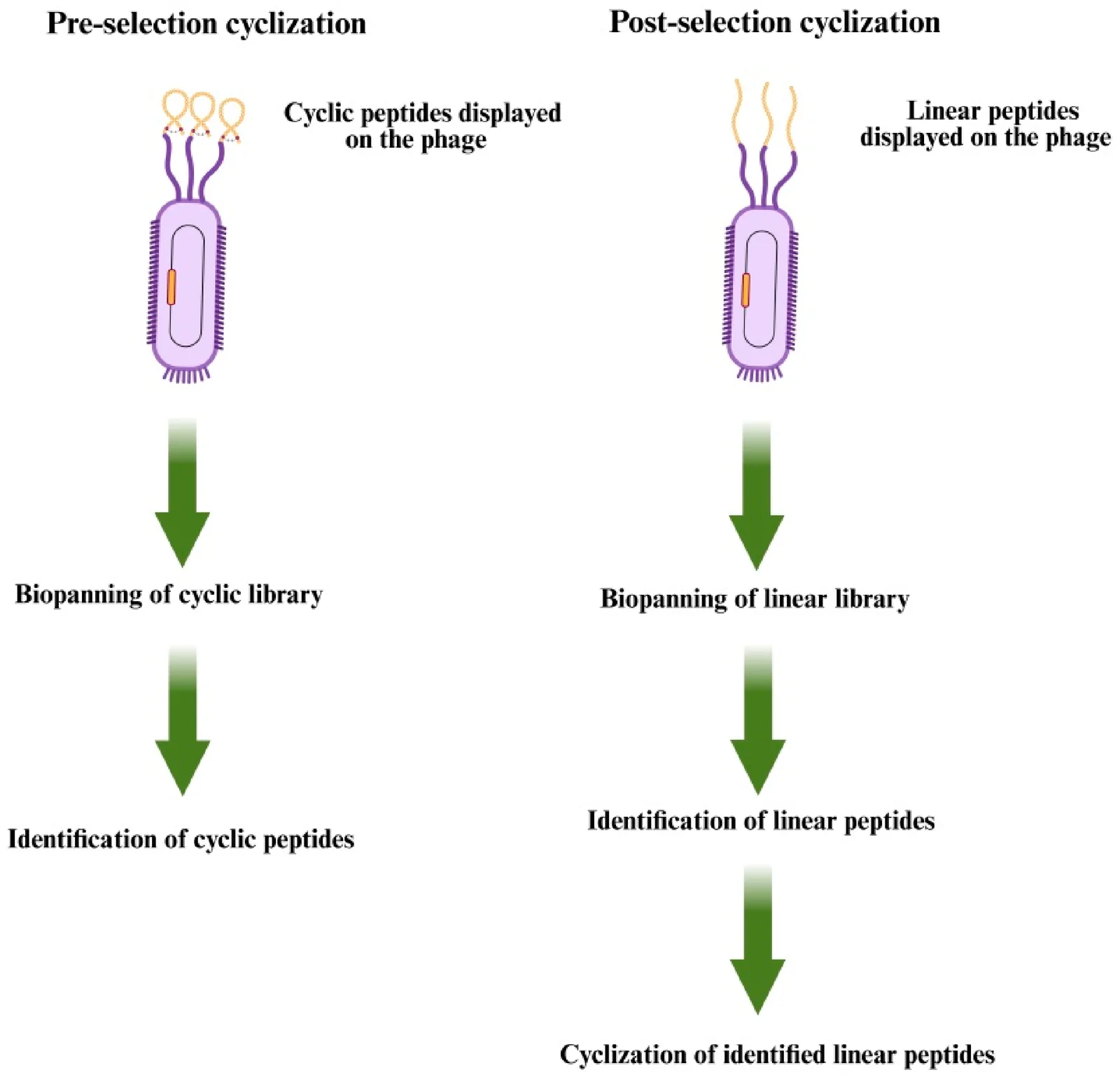
Pre-selection and post-selection cyclization of peptides within the context of phage display. In pre-selection cyclization, peptides containing cyclization-enabling residues are encoded by the phage genome and then displayed on the phage. The generated cyclic peptide library is biopanned against the target of interest, and target-binding cyclic peptides are identified. In post-selection cyclization, linear peptides are encoded by the phage genome and displayed on the phage. After biopanning of the linear phage display peptide library on the target of interest, the candidate target-binding linear peptides are cyclized either chemically or enzymatically and undergo further investigation.

In the context of phage display, peptide cyclization strategies can be subdivided based on when cyclization occurs (Figure 3). (1) Pre-selection cyclization: The amino acid residues responsible for peptide cyclization are genetically encoded by the phage genome. As a result, peptides are displayed in a cyclic form on the phage, and selection is performed directly on cyclic peptides. Genetically encoded phage-displayed cyclic peptides are mainly generated by inserting cysteine codons in the sequence encoding displayed peptides. During phage morphogenesis inside the host bacterial cells, cyclic peptides are expressed and displayed as fusions to the phage coat protein. (2) Post-selection cyclization: Peptides are originally displayed on the phage in a linear form, and phage-displayed linear peptides then undergo selection to isolate high-affinity binders for the target. After biopanning, the most promising hits identified through a variety of binding characterization studies are chemically (e.g., amide bond formation and lactam bridges) or enzymatically (e.g., sortase-mediated ligation) modified to induce cyclization. Chemically or enzymatically modified cyclic peptides are frequently generated through head-to-tail, head-to-side chain, side chain-to-side chain, and side chain-to-tail cyclization reactions [110]. Head-to-tail cyclization, also known as backbone cyclization, removes the polar N-terminal amine and C-terminal acid groups and thus reduces their negative impact on the cell membrane permeability of peptides [124,125].

Recent methodological advances in the library design and cyclization chemistries have further expanded the structural diversity, chemical space, and functional potential of phage-displayed cyclic peptide libraries. Among these advances, the development of a 100-billion-member phage display library comprising mono- and bicyclic peptides represented a major step toward expanding the library size and structural diversity accessible by phage display [126]. Containing 273 different cyclic peptide backbones, this library could provide an unprecedented skeletal diversity among phage-displayed cyclic peptide libraries. Selection of this library against human coagulation factor XIa (FXIa), an important thrombosis-associated target, enabled the identification of bicyclic peptides with nanomolar binding affinities. Notably, the isolated bicyclic peptides exhibited approximately 1000-fold higher affinity than linear peptides previously obtained against the same target region from smaller libraries, highlighting the importance of library size and structural diversity in accessing high-affinity cyclic peptide ligands. Whereas the above study highlights the importance of library architecture and skeletal diversity, recent innovations in cyclization chemistry have expanded the repertoire of macrocyclic peptide scaffolds accessible by phage display. Consistent with this, an *ortho*-phthalaldehyde (OPA)-mediated cyclization strategy has been developed that enables the rapid generation of genetically encoded cyclic peptide libraries on phage under mild physiological conditions [127]. Using this approach, a phage-displayed cyclic peptide library (with ~10^9^ members) with rigid asymmetric scaffolds was constructed and successfully screened against three therapeutically relevant targets, including protein tyrosine phosphatase 1B (PTP1B), NIMA-related kinase 7 (NEK7), and Homo Kelch-like ECH-associated protein 1 (hKeap1). The identified macrocyclic peptides exhibited micromolar binding capacity, and the PTP1B-binding peptide inhibited dephosphorylation activity while engaging a binding mode distinct from that of conventional small-molecule inhibitors, demonstrating the utility of this cyclization strategy for the discovery of functional macrocyclic peptide ligands. Building upon this work, the same group subsequently developed an enzyme-mediated cyclization approach based on tyrosinase-catalyzed o-quinone-cysteine coupling for the generation of phage-displayed macrocyclic peptide libraries [128]. Unlike chemically mediated cyclization approaches, the high selectivity and biocompatibility of tyrosinase enabled one-step peptide cyclization without compromising phage infectivity. This platform was successfully applied to identify macrocyclic peptide inhibitors against therapeutically relevant targets, including those described in their earlier OPA-mediated cyclization study, with the identified ligands exhibiting submicromolar binding affinities. This work demonstrates the potential of enzyme-mediated cyclization for phage display-based peptide discovery. Another important development has been the combination of genetic encoding via phage display with chemical modification to generate bicyclic peptides. In line with this, a new dimension was added to the exploitation of organic chemistry-based cyclization methods to enhance the properties of genetically encoded peptide libraries by developing bicyclic peptide libraries on phages [129]. The development of bicyclic peptides has provided new opportunities for the therapeutic potential of peptide ligands in targeting challenging proteins. Bicyclic peptides contain two covalent ring structures that enhance structural rigidity. With an additional cyclic constraint, bicyclic peptides offer advantages over monocyclic peptides for developing promising drug discovery scaffolds, as they exhibit more capacity for extensive interactions with globular proteins and therefore are more efficient at blocking PPIs. To achieve on-phage bicyclic peptides, a phage display library with three cysteine residues interspersed with two hexapeptide random sequences was treated with the small organic compound tris-(bromomethyl)benzene (TBMB), which is used as a scaffold to anchor phage-displayed peptides and reacts with cysteine residues to generate bicyclic peptide structures. This library was employed to identify potent and specific bicyclic peptide inhibitors of human protease plasma kallikrein with binding affinities in the nanomolar range that were further improved by affinity maturation [129]. The modification of peptides on phages in this way can expand the chemical space of phage display libraries. The same strategy was also used to isolate a high-affinity 17-mer chemically constrained bicyclic peptide with a large interaction interface that could specifically inhibit human urokinase-type plasminogen activator (uPA). Structural analysis revealed an extended conformation with multiple hydrogen bonds and complementary electrostatic interactions between both rings of the peptide and the target molecule [130].

### 5.2. Incorporation of Non-Canonical Amino Acids

With the rare exceptions of pyrrolysine and selenocysteine, the genetic code of all known organisms is constructed from the same repertoire of 20 canonical (standard) amino acids. However, this restricted and evolutionarily conserved set of amino acid building blocks imposes limitations on the structural and functional diversity achievable in natural proteins. Non-canonical amino acids (ncAAs), also known as non-standard amino acids, are amino acids that differ from the 20 canonical amino acids encoded by the universal genetic code. These molecules are naturally occurring (e.g., post-translationally modified amino acids) or synthetically designed and often have unique side chains with reactive groups and structural properties not found in the canonical amino acid repertoire. ncAAs expand the chemical and functional diversity of peptides and proteins beyond what is available through the natural genetic toolkit. Over 200 ncAAs have been genetically encoded in a variety of prokaryotic and eukaryotic organisms [131].

The incorporation of ncAAs is a powerful approach that enables the rational optimization and modification of peptides to achieve desirable structures and novel functionalities. It also holds great potential to facilitate other types of peptide modifications. For example, ncAAs can be used to enable cyclization, N-methylation, introduction of β-amino acids, stapling, and introduction of D-amino acids. Methods for incorporating ncAAs into peptides can be classified into two main categories: synthetic chemistry and in vivo biosynthetic (genetic code expansion, also known as GCE, or reprogramming) approaches. Peptides identified by phage display can be chemically synthesized with ncAAs inserted into specific sites. Alternatively, the chemical synthesis of peptides with canonical amino acids can be followed by site-specific enzymatic modifications to convert certain amino acids into a non-canonical format [132]. Despite being beneficial, these approaches are limited to short peptides, yield high costs, require complex synthetic efforts, and have restricted enzyme specificity. To address these limitations, in vivo biosynthetic approaches are used that rely on modifying the translational machinery to incorporate ncAAs into peptides during translation of the genetic code. Therefore, the power of biological translation systems is harnessed to generate peptides containing ncAAs. The in vivo incorporation of ncAAs can be achieved by using the natural translational machinery (wild-type aminoacyl-tRNA synthetases) to mischarge tRNAs and, thus, add amino acid analogues into peptides. In this strategy, the incorporation of ncAAs does not occur in a site-specific manner. However, the more precise and versatile strategy is based on utilizing the engineered translational machinery of cells to incorporate ncAAs at defined positions of a growing polypeptide chain during translation. This relies on using orthogonal aminoacyl-tRNA synthetases to perform genetic code expansion or reprogramming [15]. In genetic code expansion, ncAAs are introduced into peptides without altering the existing codon assignments, whereas genetic code reprogramming involves the reassignment of existing codons to new amino acids (e.g., reassigning sense codons to ncAAs) by radically engineering the translational machinery [133,134]. To achieve the successful incorporation of ncAAs into peptides by using the engineered strategy, four key components are required: an ncAA with tailored physicochemical attributes, a unique codon (e.g., the amber stop codon UAG or a quadruplet codon) to specify the ncAA, an orthogonal tRNA that suppresses the unique codon without cross-reactivity with endogenous tRNAs, and an orthogonal aminoacyl-tRNA synthetase that exclusively charges the ncAAs onto the orthogonal tRNA while avoiding interactions with native synthetase/tRNA pairs [135].

**Figure 4:**
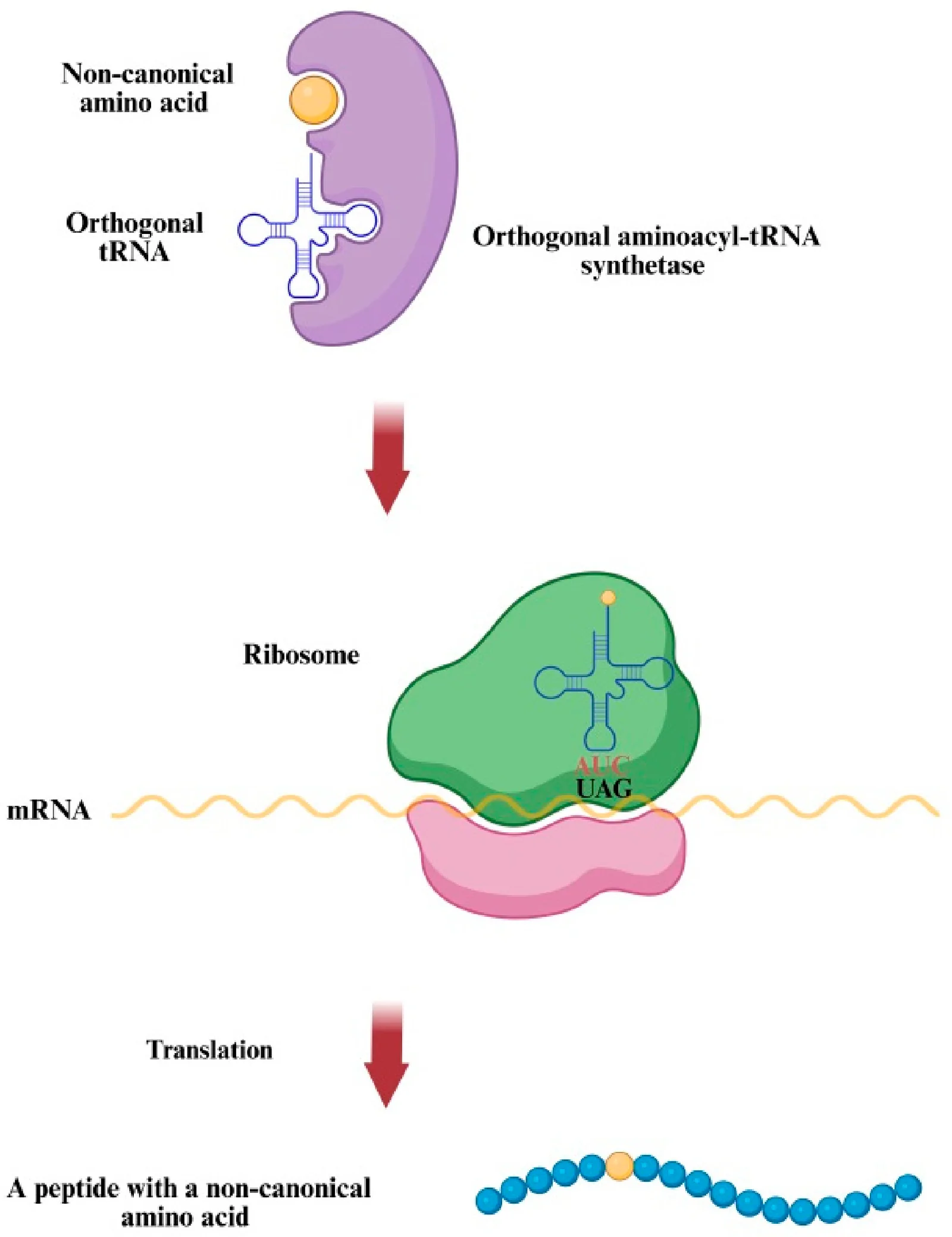
Genetic code expansion in bacterial cells by incorporating a non-canonical amino acid (ncAA) into phage-displayed peptides using orthogonal tRNA and orthogonal aminoacyl-tRNA synthetase. In the host bacterial cell, an orthogonal aminoacyl-tRNA synthetase specifically charges an orthogonal tRNA with the desired ncAA. This engineered tRNA recognizes an amber stop codon (UAG) in the mRNA (encoding the fusion phage coat protein) through its anticodon (CUA). During translation at the bacterial ribosome, the ncAA-charged orthogonal tRNA binds to the stop codon, suppresses it, and inserts the ncAA into the growing peptide chain. As translation proceeds, the synthesized peptide displayed on the phage surface contains the incorporated ncAA. This strategy enables the site-specific incorporation of ncAAs into phage-displayed peptides.

Compared to in vitro cell-free display systems, cell-dependent display approaches have considerable limitations for incorporating ncAAs into displayed peptides. In phage display, these limitations originate from the biological constraints associated with crosstalk between phage particles and host bacterial cells. Phage particles are dependent on their host bacteria for protein expression. However, bacterial cells lack the native translational machinery to efficiently incorporate diverse ncAAs into phage proteins during translation. To address this challenge and apply genetic code expansion to phage display, bacterial cells with engineered tRNA and aminoacyl-tRNA synthetase pair are needed [136]. Figure 4 illustrates the incorporation of an ncAA into a peptide using the orthogonal pair of tRNA and aminoacyl-tRNA synthetase. Genetic code expansion in phage display is largely based on recoding the amber stop codon (UAG) to make it recognizable by a mutant suppressor tRNA that can be charged with an ncAA. For this purpose, an amber codon is inserted into the target site in the coding region of the displayed peptide library, where the ncAA is going to be incorporated. The expression of an engineered tRNA containing an anticodon (CUA) that can base pair with the UAG codon in the mRNA in the bacterial cell results in stop codon suppression; the ribosome then continues to synthesize the peptide, and the ncAA carried by the engineered tRNA will form a peptide bond with the growing peptide in the ribosome [137]. The culture medium is supplemented with the ncAA, which is taken up by bacterial cells and utilized by the engineered aminoacyl-tRNA synthetase to charge the suppressor tRNA. Phage particles secreted from bacterial cells present the displayed peptide containing the ncAA when the stop codon is successfully suppressed. The incorporation of ncAAs into peptides displayed on the phage expands the chemical diversity of phage combinatorial libraries and provides novel opportunities for phage display-based ligand discovery. In support of this notion, phage display with expanded genetic code has been used to incorporate various ncAAs into displayed peptides, allowing for diverse functional applications, such as bioconjugation [138], binding to metal ions [139], metal substitution in DNA-binding domains [140], and cyclization [141]. Furthermore, recent advances in the site-specific incorporation of various ncAAs into phage-displayed peptides have demonstrated that these amino acids are not merely tools for expanding chemical diversity but also create new possibilities for engineering peptide libraries with enhanced structural complexity, improved stability, and novel functional properties that are difficult or impossible to obtain using the canonical amino acid repertoire alone. Consequently, this strategy has evolved into a versatile platform for generating phage-displayed peptide libraries tailored for the discovery of peptide ligands with improved pharmacological properties. Several recent studies illustrate how ncAA incorporation has been successfully exploited to engineer phage-displayed peptide libraries with enhanced structural and functional properties. For example, a genetically encoded propiolamide-bearing ncAA was developed to construct cyclic peptide libraries through proximity-induced crosslinking between the electrophilic propiolamide warhead and a neighboring cysteine residue, producing stable vinyl sulfide-linked macrocycles [142]. Site-specific incorporation of the ncAA using amber codon suppression enabled efficient construction of recombinant phage libraries without compromising phage display, and biopanning identified cyclic peptide inhibitors targeting the SARS-CoV-2 main protease. This work expanded the limited repertoire of electrophilic ncAAs available for phage display and demonstrated their utility for discovering therapeutically relevant cyclic peptide ligands. In another recent study, a lysine-selective photocyclization strategy was introduced using the genetically encoded ncAA *o*-nitrobenzyl alcohol lysine (o-NBAK) [143]. Following incorporation into phage-displayed peptides, mild photoactivation triggered efficient intramolecular cyclization with a neighboring lysine residue to generate stable indazolone macrocycles while preserving phage infectivity. Screening of the cyclized libraries against disease-relevant protein targets yielded cyclic ligands with nanomolar to low-micromolar binding affinities, and macrocyclization increased proteolytic stability by more than one order of magnitude, highlighting the potential of ncAA-enabled cyclization for improving peptide pharmacological properties. A further recent application exploited the incorporation of *N*ε-butyryl-L-lysine into phage-displayed peptide libraries to direct selections toward epigenetic reader proteins that specifically recognize lysine butyrylation [144]. This strategy enabled the discovery of peptide ligands beyond conventional histone-derived substrates and provides a versatile platform for developing selective probes and potential inhibitors targeting epigenetic readers, writers, and erasers implicated in numerous human diseases. Taken together, these studies demonstrate that the integration of ncAAs into phage display extends well beyond expanding sequence diversity. As the repertoire of genetically encoded ncAAs continues to expand, their integration with phage display is expected to further accelerate the discovery of next-generation peptide ligands possessing enhanced pharmacological properties and functionality, broadening their therapeutic applicability.

Despite these remarkable advances, several challenges continue to limit the broader application of ncAA incorporation into phage-displayed peptide libraries. These include low efficiency of amber suppression, reduced fidelity of the orthogonal tRNA-aminoacyl tRNA synthetase pair, reduced phage yield and fitness associated with the metabolic burden of the GCE machinery on cells, limited codon availability, and toxicity or low membrane permeability of some ncAAs, all of which can lead to compromised library complexity and diversity as well as reduced efficiency in selecting ncAA-containing peptides. An effective alternative modality for incorporating ncAAs into peptides is to use cell-free in vitro translation (IVT) systems, which provide greater flexibility in the type of incorporated ncAAs and offer the ability to generate libraries with larger diversity compared to cell-dependent translation platforms like phage display. PURE (Protein synthesis Using Recombinant Elements) is a popular reconstituted IVT system [145]. FIT (Flexible In vitro Translation) is a specialized system based on PURE that combines PURE with custom aminoacylation of tRNAs using flexizymes. Flexizymes are a group of promiscuous aminoacylating ribozymes that essentially recognize activated carboxylates [146]. Due to being engineered for minimal specificity toward the discriminatory nucleotides in tRNAs, flexizymes recognize a diverse range of tRNAs as substrates and, thus, are capable of charging tRNAs with a wide range of amino acids, including many ncAAs, without requiring aminoacyl-tRNA synthetases. By possessing this unique functionality, flexizymes bypass the natural specificity and restrictions of the standard translation machinery and thus enable extensive genetic code reprogramming [147,148]. Currently, FIT represents one of the most versatile and efficient platforms, in particular for genetic code reprogramming, in cell-free environments. The integration of the FIT system with mRNA display, as an in vitro display methodology, has led to the development of the random non-standard peptide integrated discovery (RaPID) system [149,150]. Thanks to its adaptability and flexibility, RaPID has become a widely used tool in drug discovery for generating diverse, high-affinity macrocyclic peptides against different target proteins previously considered undruggable. Companies such as PeptiDream have commercialized this platform, both independently and in collaboration with global pharmaceutical partners, to develop an extensive pipeline of peptide-based diagnostics and therapeutics. The peptide candidates discovered by using RaPID are in different stages of clinical development and target various clinically relevant proteins. Among clinically validated peptides discovered using related technologies, the macrocyclic peptide Zilucoplan, originated by Ra Pharmaceuticals and later developed by UCB, is now on the market and was FDA approved in 2023 for the treatment of an autoimmune disease called generalized myasthenia gravis (gMG) [151,152]. This peptide drug was developed through a targeted screen using the innovative Extreme Diversity mRNA display platform [153]. This 15-amino acid peptide drug is a complement component 5 (C5) inhibitor. It binds to the C5 protein and prevents its cleavage into C5a and C5b. This biological function inhibits the formation of the membrane attack complex, which in turn protects the postsynaptic membrane from damage and maintains proper neuromuscular transmission. By targeting the complement system, Zilucoplan addresses a key driver of the autoimmune response in gMG and provides a novel therapeutic approach for patients, in particular those who are positive for anti-AChR (anti-acetylcholine receptor) antibodies [154]. This drug has also been investigated in preclinical evaluations or clinical trials for several additional conditions, such as immune-mediated necrotizing myopathy (IMNM), amyotrophic lateral sclerosis (ALS), paroxysmal nocturnal hemoglobinuria (PNH), lupus nephritis (LN), and other autoimmune diseases that may benefit from complement inhibition.

### 5.3. Introduction of D-Amino Acids and Mirror Image Phage Display

**Figure 5:**
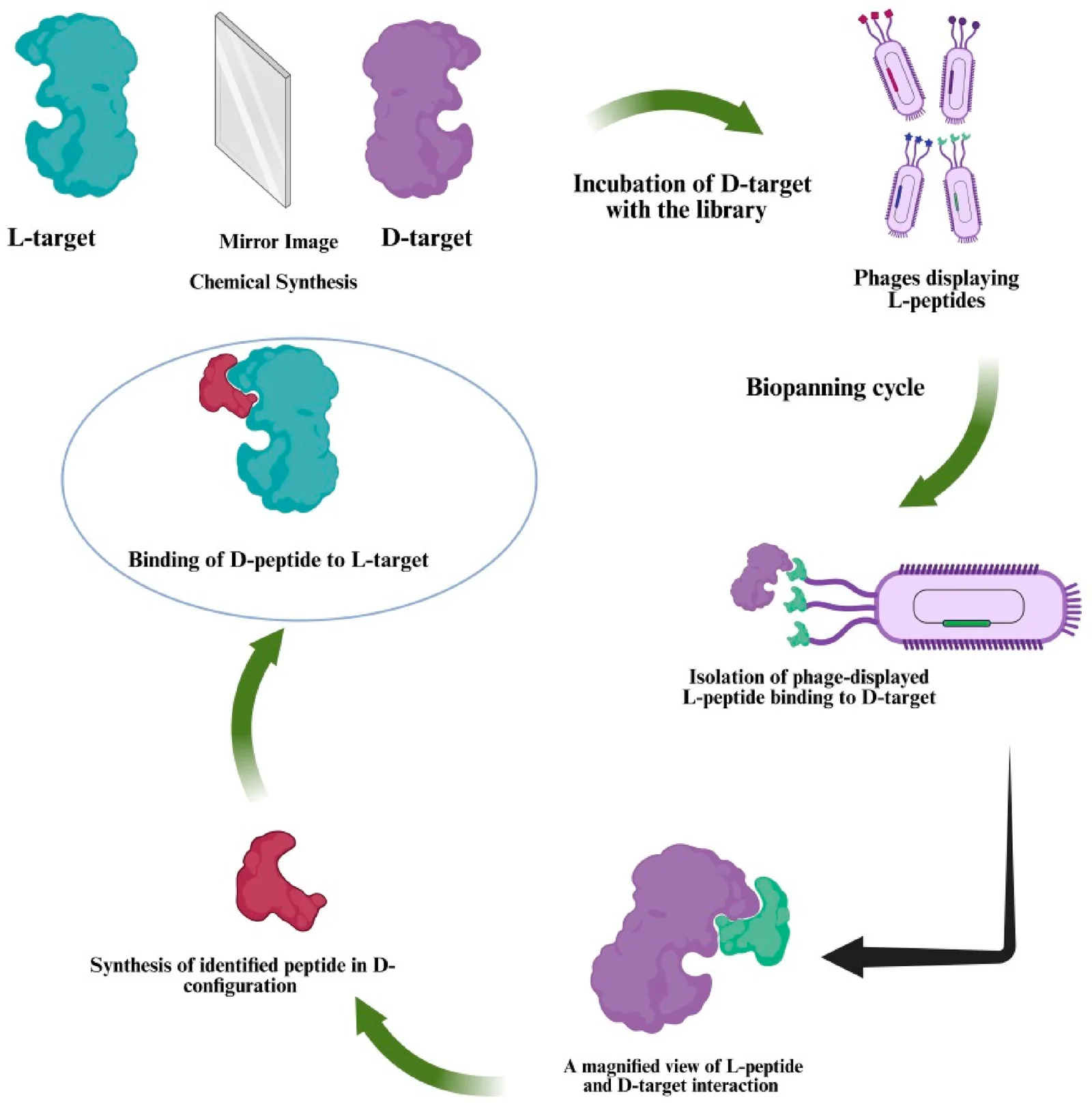
Mirror image phage display. In the first step, the mirror image (D-enantiomer) of the target L-protein is chemically synthesized to produce the D-target. A phage display library containing L-peptides is then incubated with the D-target to enable binding interactions. Through iterative biopanning cycles, phages displaying L-peptides that specifically bind to the D-target are enriched and isolated. A magnified view (bottom right) illustrates the interaction between the selected phage-displayed L-peptide and the D-target. The identified L-peptide sequences are then chemically synthesized in their D-enantiomeric form. The resulting D-peptides are capable of binding to the original L-target protein.

Except for glycine, all proteinogenic amino acids are chiral molecules that exist as either L- or D-configurations, with L-amino acids being predominantly used in nature for the biological synthesis of proteins. D-proteins are mirror images of natural L-proteins. Due to differences in backbone and side chain geometry, D-proteins are not efficiently recognized by L-proteins such as natural proteases. This characteristic renders D-proteins more resistant to proteolytic degradation, highlighting the potential of introducing D-amino acids into the peptide backbone as a potent strategy to enhance its stability in biological environments [155]. The resistance of D-peptides to proteolytic degradation substantially increases their half-life and stability in serum with longer circulation times and elevated bioavailability, which is particularly important for the development of therapeutics. D-enantiomeric peptides are also generally less immunogenic, as they are inefficiently processed and presented by antigen-presenting cells (APCs). A smart approach to obtain target-binding peptide ligands in D-configuration is mirror image phage display (Figure 5). This technique is a variation of conventional phage display but involves the chemical synthesis of the target of interest with D-enantiomeric amino acids (a mirror image of the original target molecule). This target is then used to identify binders from a phage library displaying L-peptides, followed by chemical synthesis of the selected L-peptide(s) with D-amino acids. The resulting D-peptides can interact with the original target protein (containing L-amino acids) [156].

The chemical synthesis of target molecules in D-configuration, in particular large proteins, represents a barrier for mirror image phage display, leading this technique to rely mainly on peptides and small proteins, such as HIV-1 gp41-derived peptides and amyloid peptide Aβ, as the target of library selection. In this regard, mirror image phage display was employed to identify a D-peptide antagonist that could bind to the hydrophobic pocket in the N-peptide coiled-coil region of gp41 (a key domain of the envelope glycoprotein of HIV-1). The resulting D-peptide exhibited potent antiviral activity by blocking gp41-mediated membrane fusion and inhibiting viral entry [157]. Also, this technique was used to select a D-peptide antagonist that could bind to the aggregation-prone amyloid peptide Aβ, demonstrating the potential of D-peptides as protease-resistant modulators for targeting amyloid aggregates in Alzheimer’s disease [158]. More recently, mirror-image phage display was used to identify cyclic all-D-peptides targeting C–X–C motif chemokine ligand 8 (CXCL8, IL-8), a key regulator of neutrophil recruitment and angiogenic responses implicated in inflammation and the progression of diseases such as colorectal cancer, HIV-associated neurocognitive disorder, and psoriasis [159]. The selected D-peptides exhibited submicromolar binding affinities toward CXCL8 and modulated chemokine function by disrupting the native CXCL8 dimer and inhibiting its interaction with glycosaminoglycans (GAGs), a critical step in chemokine signaling. Although further structural optimization is required to achieve therapeutic-level potency, this study highlights the potential of protease-resistant D-peptides as chemokine-neutralizing agents.

Despite limitations for the production of D-proteins, technological advancements in peptide and protein synthesis by developing methods, such as the native chemical ligation technique, have allowed for the synthesis of larger domain-sized proteins, significantly enhancing the scope, utility, and effectiveness of mirror image phage display in peptide-based therapeutic development [160]. In a parallel development, a scalable approach based on automated fast-flow peptide synthesis (AFPS) was established for the rapid production of mirror-image D-protein targets, overcoming a major limitation associated with the low-throughput preparation of synthetic D-proteins [161]. This platform enabled the discovery of six macrocyclic D-peptide ligands against the oncoprotein MDM2 and the E3 ubiquitin ligase CHIP. Structural analysis revealed that the identified D-peptides engaged their targets through defined binding interactions, while the MDM2-targeting ligands exhibited micromolar binding affinities and CHIP-targeting ligands bound recombinant CHIP and displaced a native ligand at low micromolar concentrations, highlighting the potential of mirror-image phage display for generating stable peptide modulators of therapeutically relevant protein interactions.

The pharmacological characteristics of peptides can also be improved by chemically synthesizing retro-inverso (RI) analogues of L-peptides, also known as retro-D-peptides. RI peptides are composed entirely of D-amino acids, but their primary sequence is reversed (i.e., from C-terminus to N-terminus). Compared to L-peptides, D-peptides often adopt different secondary and tertiary structures that can interfere with their capacity to interact with biological targets. However, RI peptides better preserve side-chain topology and structural motifs, allowing them to potentially retain biological activity of the original L-peptide while still being protease-resistant [162,163]. To find suitable binders, D-peptides require specialized selection techniques like mirror image phage display, whereas RI peptides can be derived more directly from known bioactive peptides, reducing the time and effort for peptide discovery and optimization.

### 5.4. Oligomerization

As already noted, many peptides derived from phage display, in particular cell-targeting peptides, exhibit poor affinity and activity when synthesized as free monomeric peptides out of their original phage context. The affinity and biological activity of peptides can be improved by oligomerization, where monomeric peptides are linked together to form dimers, trimers, tetramers, or higher oligomeric forms [89,164]. Oligomerization of peptides can be achieved through non-covalent interactions and covalent bonding. Non-covalent self-assembly is the natural ability of some peptides to form functional oligomers or multimers through hydrophobic interactions (amphiphilic peptides), electrostatic interactions (charged peptides), β-sheet stacking, and α-helix assembly. However, in covalent bonding, monomers are linked through direct peptide bond formation (via chemical amide bond formation or enzymatic ligation), disulfide bridges, and chemical crosslinking (using agents such as glutaraldehyde, maleimides, and PEG-based spacers). Trilysine has been demonstrated to be an effective scaffold for covalently multimerizing cell-targeting peptides selected from phage display libraries and preserving the peptide activity outside the phage framework. Trilysine mimics the valency and orientation of peptides displayed on the pIII of the M13 phage. In addition, it can be modified with a range of different moieties, such as drugs, drug carriers, biotin, dyes, and metal chelators, without substantially influencing peptide binding [40]. Consistent with this, the trilysine-mediated tetramerization of a peptide that binds to the integrin α_v_β_6_-expressing lung adenocarcinoma cells was shown to significantly enhance its affinity for the target cell compared to the monomeric peptide [165,166]. Multimericity can also increase the serum half-life of peptides due to decreased susceptibility to proteases [167]. In addition, dimerization has been successfully employed as an affinity maturation strategy for phage display-derived peptides. A HER2-binding peptide identified by phage display was converted into a homodimer using a hairpin-structured peptide linker, improving its binding affinity from a Kd of 4.1 × 10^−7^ M to 2.2 × 10^−10^ M, comparable to that of the anti-HER2 antibody trastuzumab [168]. Given the overexpression of HER2 in several cancers, this study highlights the potential of peptide dimerization to generate antibody-mimicking ligands with diagnostic and therapeutic applications. Beyond affinity maturation, dimerization has also been exploited to enhance the targeting performance of phage display-derived peptides. A dimeric IGF2R-binding peptide, a ligand for a receptor overexpressed on activated hepatic stellate cells in liver fibrosis, was used to functionalize a peptide-based siRNA delivery system [169]. The dimeric peptide significantly enhanced cellular uptake and transfection efficiency in activated hepatic stellate cells, while an in vivo biodistribution study in a rat model of liver fibrosis demonstrated preferential accumulation of the therapeutic nanocomplex in the fibrotic liver, demonstrating the capacity of peptide dimerization for improving targeted delivery and therapeutic efficacy.

It is of interest to note that receptor density on the surface of the target cell is a contributory factor in improving the affinity of multimeric peptides, and the existence of a larger number of receptor molecules raises the likelihood of synergistic improvement in peptide apparent affinity. This emphasizes the significance of optimizing affinity through multimerization in the context of whole cells, where the natural receptor landscape is preserved. One subcategory of peptide oligomerization is peptide concatamerization [170]. Oligomeric peptides can be linear, cyclic, or branched. However, a peptide concatamer is a linear, repeated peptide sequence formed by chemical synthesis or, more commonly, genetic fusion. The repeating units in a peptide concatamer are linked together through covalent bonds. Overall, recent advances in peptide oligomerization and multimerization have further expanded its therapeutic potential by enabling the design of increasingly sophisticated multivalent architectures with improved target specificity, binding affinity, and pharmacokinetic properties. By enhancing peptide performance, multimeric constructs are being explored for targeted drug delivery, molecular imaging, antimicrobial therapy, and vaccine development. The ability to engineer complex branching structures that mimic or interfere with natural molecular interactions also offers exciting opportunities for the development of safer, more effective, and increasingly selective peptide therapeutics [164].

### 5.5. Chemical Conjugation and Genetic Fusion

Conjugation to other molecules to increase the hydrodynamic radius and molecular weight of peptides, as well as address the pharmacologic limitations associated with the small size of these molecules, is a common strategy in peptide drug development. This approach is an efficient means to improve the pharmacological properties of peptides by prolonging half-life, enhancing protection against proteolytic enzymes, and overcoming rapid clearance from the body. PEGylation is a widely used conjugation strategy that involves attaching polyethylene glycol (PEG) chains to peptides. PEG is a non-toxic polymer with low immunogenicity that shields peptides against degradation by proteases via enhanced steric hindrance and enhances water solubility, thereby improving the pharmacokinetic properties of peptides [171]. Conventional PEGylation typically targets lysine or cysteine amino acids. When multiple reactive lysine or cysteine residues are present in a peptide, nonspecific conjugation can occur at different sites randomly, resulting in a heterogeneous mixture of conjugation products that are difficult to purify and characterize. Variations in the degree of PEGylation and the resultant polydispersity of PEGylated products are less challenging for short peptides. To address this issue for long peptides, site-specific PEGylation techniques are used that enable the precise attachment of PEG moieties to peptides with selectivity and positional control [16,172,173]. Pegcetacoplan is an example of an FDA-approved PEGylated peptide available in the drug market. Pegcetacoplan is a PEGylated cyclic peptide drug that acts as an inhibitor of complement C3 and is primarily used for the treatment of PNH and geographic atrophy (GA) [174]. One notable example of PEGylation-mediated optimization of phage display-derived ligands involved the CAQK peptide, which was identified through in vivo phage display in a mouse model of traumatic brain injury (TBI) and targets injury-associated extracellular matrix components, including tenascin-C [175]. Although CAQK exhibits therapeutic potential by promoting neural repair and functional recovery after TBI, its rapid systemic clearance limits clinical translation. To overcome this limitation, CAQK was conjugated to a PEG scaffold through two different linker chemistries, generating long-circulating peptide–polymer conjugates. PEGylation extended the blood half-life of CAQK by approximately 90-fold and significantly enhanced accumulation of the peptide in injured brain regions in a mouse model of TBI. Both PEG conjugates showed colocalization with tenascin-C in the perilesional cortex and prolonged peptide retention within injured brain tissue for 24 h. Importantly, linker chemistry influenced biodistribution, with maleimide-linked conjugates exhibiting reduced accumulation in filtration organs compared with DBCO-linked conjugates. In a prophylactic dosing study, the optimized CAQK–PEG conjugate achieved 4.5% injected dose per gram of peptide accumulation in injured brain tissue 24 h after administration. These findings demonstrate that PEGylation can substantially improve the pharmacokinetic profile and tissue targeting of phage display-derived peptides, underscoring the importance of conjugation chemistry in developing peptide-based therapeutics. Lipidation is another strategy to improve the pharmacological features of peptides in which lipid moieties (e.g., fatty acids) are covalently attached to peptides. The lipid molecules can bind to serum albumin, resulting in reduced degradation and diminished clearance rate of peptides, as well as their slower release from the injection site. In addition, lipidation facilitates interaction with cell membranes and may enhance permeation across biological barriers [176]. The GLP-1 receptor agonists liraglutide and semaglutide are examples of peptide drugs conjugated to a fatty acid chain (palmitoyl group and C18 fatty diacid, respectively) that are used for the treatment of type 2 diabetes [177]. A representative example of lipidation-mediated optimization was reported for the phage display-derived PCSK9 inhibitory peptide P9, which targets proprotein convertase subtilisin/kexin type 9 (PCSK9), a clinically validated regulator of cholesterol metabolism [178]. Following sequence optimization to improve proteolytic stability, the peptide was conjugated to a short lipidated albumin-binding tag, generating P9-alb fusion peptides with high affinity for serum albumin. Although lipidation resulted in a modest reduction in PCSK9 binding affinity, the conjugates retained nanomolar inhibitory activity (~40 nM) while exhibiting markedly improved pharmacokinetic properties. Albumin binding increased the peptide half-life in human serum from 7 h to more than 48 h and extended the in vivo half-life in mice approximately 10-fold (40.8 vs. 3.6 min) by reducing proteolytic degradation and renal clearance. Importantly, the enhanced stability translated into superior in vivo efficacy, with improved low-density lipoprotein receptor (LDLR) recovery and greater cholesterol-lowering activity than the unconjugated peptide, despite its lower in vitro affinity. At higher doses, the optimized peptide achieved cholesterol-lowering effects comparable to those of the clinically approved anti-PCSK9 monoclonal antibody evolocumab. This study suggests that improving pharmacokinetic properties through lipidation and albumin binding can be more critical for therapeutic performance than maximizing binding affinity alone.

Two plasma proteins, serum albumin and immunoglobulin G (its Fc region), with exceptionally long circulation times, are also employed to extend half-life and prolong the duration of action of peptides through recombinant fusion [11]. This type of modification has been applied to drugs such as albiglutide, dulaglutide, and romiplostim. The GLP-1 receptor agonists albiglutide (withdrawn from the market due to commercial reasons, not safety issues) and dulaglutide, both developed for the treatment of type 2 diabetes, are peptide drugs fused to the human serum albumin and the Fc fragment of IgG, respectively [179,180]. Romiplostim is a thrombopoietin receptor agonist peptide fused to the Fc region of IgG1 that is used to treat chronic immune thrombocytopenic purpura (ITP) [181].

Taken together, these chemical conjugation and genetic fusion strategies have become indispensable tools in peptide engineering by improving the pharmacokinetic and pharmacodynamic properties of peptide therapeutics. Continued advances in these areas are expected to further enhance the clinical translation of peptide ligands discovered through phage display.

### 5.6. Stapling

**Figure 6:**
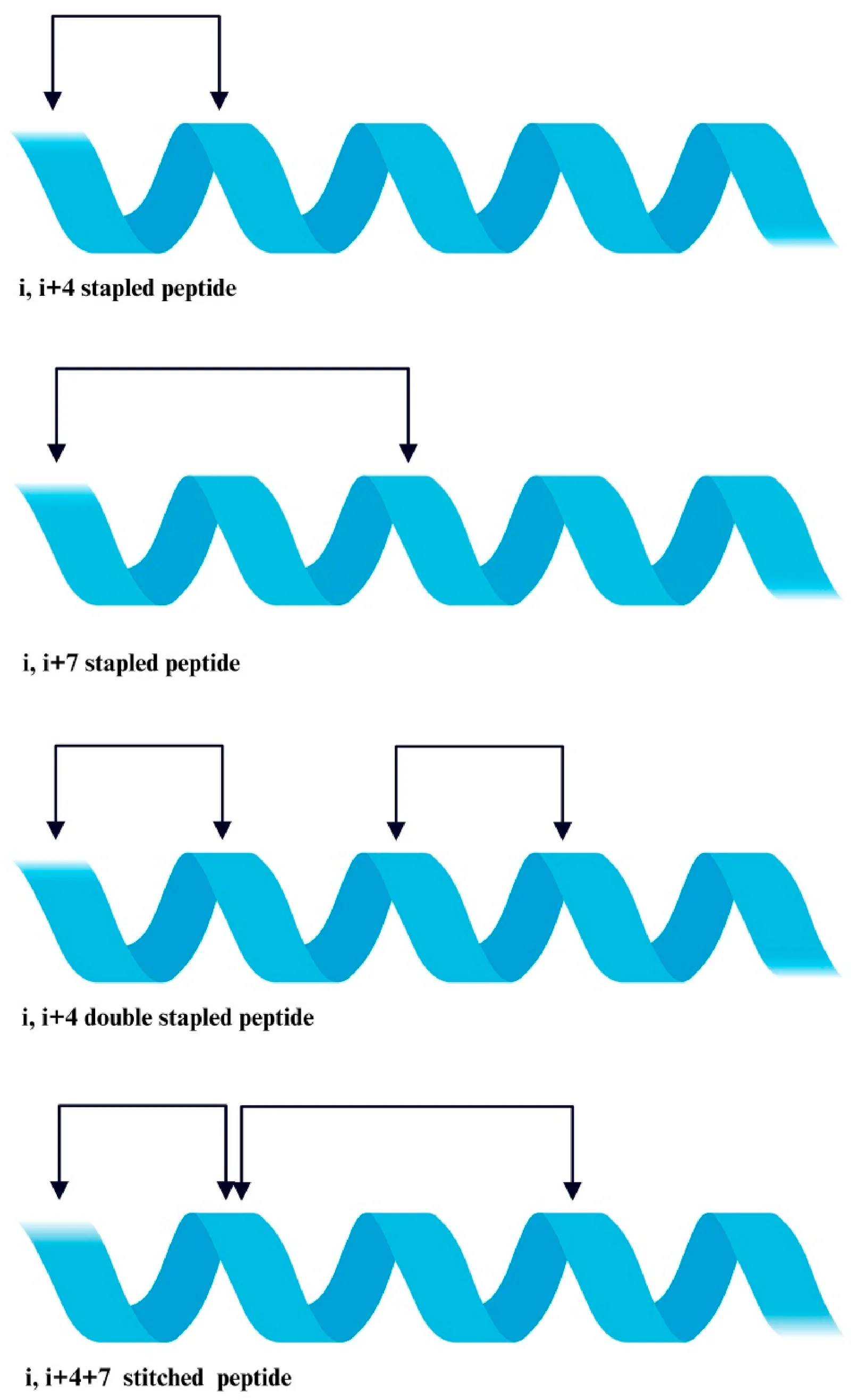
Different types of peptide stapling to stabilize α-helical structures. The top panel depicts an *i, i+*4 stapled peptide, where a single staple is introduced between residues at positions i and i+4. The second panel shows an *i, i+*7 stapled peptide, where a single staple bridges residues *i* and *i+*7. The third panel illustrates an i, i+4 double-stapled peptide, introducing two separate *i, i+*4 staples to further enhance helix stability. The fourth panel represents an i, i+4/i+7 stitched peptide, in which a single long staple spans *i, i+*4, and *i+*7 residues, effectively stitching the helix together for robust structural integrity and conformational stability. Stitching results in greater helix stabilization.

Stapling involves the rational positioning of covalent crosslinks (staples) between side chains of different amino acids within a peptide through various chemistries (e.g., hydrocarbon, lactam, and disulfide bridging) and enzymatic reactions (e.g., transglutaminase-mediated crosslinking). Stapling primarily aims to stabilize α-helical structures by introducing crosslinks between residues at positions i and i+4 or positions i and i+7, which lie on the same side of the helix (Figure 6). This crosslinking restricts the conformational freedom of the peptide backbone, favoring orientations compatible with helix formations [16]. By locking the peptide into a helical geometry due to the spatial constraints imposed by the crosslink, the intramolecular hydrogen bonds that define helices are more likely to be stabilized. Helices are among the most prevalent secondary structural elements [182], with α-helices representing the predominant helical motif and being stabilized by intramolecular hydrogen bonds [16]. By mimicking, replicating, and stabilizing α-helices in peptides, researchers can develop targeted modulators of PPIs. The stapling-mediated modification of α-helices has been demonstrated to improve cell permeability, target binding affinity, and proteolytic stability [183]. The therapeutic potential of stapling has been further demonstrated through its integration with phage display for the discovery of peptide modulators of protein–protein interactions. A phage library displaying i, i+7 stapled helical peptides was constructed by introducing cysteine residues at defined positions and chemically crosslinking them with a bifunctional linker to stabilize the helical conformation [184]. Using this platform, stapled peptides targeting the E3 ligase regulator HDM2 (a key negative regulator of the tumor suppressor p53 and an important anticancer target) were identified. The selected peptides reproduced critical hydrophobic hotspot residues required for HDM2 binding and showed the ability to disrupt the HDM2–p53 interaction. The approach was further applied to the SARS-CoV-2 spike receptor-binding domain (RBD), generating stapled peptides capable of inhibiting spike RBD–ACE2 interaction. These findings indicate the capacity of stapling strategies to generate proteolytically stable, high-affinity peptide modulators of therapeutically important protein–protein interactions. Beyond its application during peptide discovery, stapling has also been employed as a post-selection optimization strategy for phage display-derived peptide ligands. Starting from the GABARAP-binding peptide K1 identified through phage display, structure-guided stapling generated constrained peptides with nanomolar affinity toward GABARAP and improved resistance to biological degradation. The optimized stapled peptides exhibited cytosolic penetration, reduced autophagic flux in ovarian cancer cells, and enhanced the sensitivity of cancer cells to cisplatin. These peptides function as autophagy-modulating compounds and showed synergistic effects with DNA-damaging chemotherapy in ovarian cancer cells, demonstrating the importance of stapling to transform peptide ligands into pharmacologically relevant protein–protein interaction modulators [185].

Recent reviews have further highlighted the expanding applications of stapled peptides for targeting PPIs and improving the pharmacological properties of therapeutic peptides [183,186,187]. Reversible stapling strategies further extend the chemical space of peptide stapling by retaining the structural and pharmacological advantages of conventional staples while enabling controlled release of peptide molecules and other cargoes, thereby broadening the utility of peptides in chemical biology, drug delivery, and therapeutic development [188]. Currently, no stapled peptides have received FDA approval for clinical use. However, several stapled peptide candidates have advanced into clinical trials, such as ALRN-6924 (developed by Aileron Therapeutics), ATSP-7041 (developed by AstraZeneca and Aileron Therapeutics), and SAH-P53-8 (developed by Sanofi and Dana-Farber Cancer Institute), all investigated as anticancer agents [183]. Although stapled peptides are considered promising for the development of therapeutic agents, complex and costly manufacturing, potential immunogenicity, and significant delivery challenges are limitations that need to be addressed for their successful clinical translation.

### 5.7. Translational Challenges and Manufacturing Considerations in Peptide Optimization and Drug Development

Although phage display has enabled the more efficient identification of target-specific peptide ligands, the translation of these molecules into therapeutic products requires overcoming several additional challenges beyond target recognition and biological activity. As discussed in previous sections, optimization strategies can substantially improve various pharmacological properties of peptides. However, these approaches may introduce new challenges related to chemical synthesis, purification, process scalability, manufacturing costs, and regulatory requirements. Therefore, the successful development of phage display-derived peptide therapeutics necessitates not only the identification of functional peptide sequences but also consideration of their manufacturability and compatibility with industrial production workflows. Achieving this goal entails a broader development perspective in which biological performance is considered together with practical considerations associated with synthesis, process scalability, quality control, and regulatory feasibility. Early consideration of these factors during peptide optimization can facilitate downstream development, reduce technical barriers during technology transfer, and improve the likelihood that promising peptide candidates can progress from early-stage discovery to clinically and commercially viable therapeutics. In this context, peptide discovery and peptide manufacturing should not be viewed as independent stages but rather as interconnected components of an integrated drug development process.

One of the major challenges in peptide drug development is the transition from laboratory-scale synthesis and characterization to robust, large-scale manufacturing processes. While many peptide candidates can be readily synthesized at analytical or research scales, their production as active pharmaceutical ingredients (APIs) under good manufacturing practice (GMP) conditions requires extensive process optimization and control [189]. Increasing peptide length, introducing complex chemical modifications, or incorporating non-natural building blocks can significantly affect synthetic efficiency, impurity profiles, and overall production yield. In particular, therapeutic peptides often require multiple synthetic steps involving protecting-group strategies, coupling reactions, and post-synthetic modifications, each of which can contribute to reduced yield and increased process complexity [190,191]. Consequently, a peptide sequence that demonstrates excellent biological activity during discovery may not necessarily represent an optimal therapeutic candidate if its synthesis is inefficient, expensive, or difficult to scale. The chemical complexity introduced during peptide optimization can also create substantial challenges during purification and quality control. Unlike small-molecule drugs, peptide products frequently contain structurally related impurities generated during synthesis, including deletion sequences, truncated peptides, racemization products, and incorrectly modified species [189,190,192]. Efficient removal of these impurities often depends on advanced chromatographic purification approaches, which can represent a major cost-driving step in peptide manufacturing. Furthermore, purification becomes increasingly challenging as peptide size increases or when peptides contain hydrophobic residues, conformational constraints, or non-canonical chemical modifications. The development of scalable purification strategies that maintain product quality while minimizing resource consumption remains a critical aspect of therapeutic peptide manufacturing [189]. These manufacturing considerations are particularly relevant for phage display-derived peptides because the discovery process and the final production process are fundamentally different. During phage display selection, peptide sequences are generated through biological expression and amplified through replication of the encoding genetic information. However, most therapeutic peptides identified through phage display ultimately rely on chemical synthesis or advanced recombinant production approaches for clinical development. Therefore, peptide sequences selected solely based on binding affinity may need further engineering to achieve an appropriate balance between biological activity and manufacturability. For example, highly constrained cyclic peptides or peptides containing ncAAs may provide enhanced stability and potency but may also require more complex synthetic workflows. Similarly, stapled peptides can demonstrate improved conformational stability and target engagement, but the introduction of chemical crosslinkers may increase synthetic complexity and influence purification requirements. Thus, the optimization of phage display-derived peptides should consider developability parameters alongside biological performance.

Sustainability represents another increasingly important consideration in peptide manufacturing. Conventional peptide synthesis, particularly solid-phase peptide synthesis (SPPS), relies on large quantities of organic solvents, protecting-group reagents, and coupling agents, which can generate significant chemical waste compared with the amount of final peptide product obtained [191]. Solvent use and waste generation are major contributors to the environmental footprint of peptide production, particularly during large-scale manufacturing. Recent advances in peptide chemistry aim to address these challenges through improved reaction efficiency, solvent replacement, recycling strategies, automation, and alternative synthetic methodologies. Nevertheless, achieving a balance between sustainability, process efficiency, product quality, and regulatory compliance remains a major challenge for industrial peptide production. Process development and technology transfer constitute additional critical steps between peptide discovery and commercial manufacturing. A peptide synthesis method optimized in an academic or early-stage industrial setting may require substantial modification before implementation at manufacturing scale. Factors such as equipment compatibility, batch-to-batch reproducibility, raw material quality, analytical methods, and regulatory documentation must be carefully evaluated during technology transfer [189,193]. In this context, early consideration of manufacturing feasibility during peptide optimization can reduce development timelines and prevent costly redesign at later stages. The pharmaceutical industry increasingly emphasizes the concept of design for manufacturability, in which candidate selection incorporates not only potency and efficacy but also practical production requirements. Regulatory considerations further influence the development pathway of peptide therapeutics [193]. Peptide products must meet stringent specifications regarding identity, purity, potency, stability, and impurity characterization. The introduction of chemical modifications, such as ncAAs, macrocyclization strategies, or synthetic linkers, may need additional analytical characterization and regulatory evaluation. Furthermore, differences between synthetic peptide products and naturally occurring biological molecules may influence immunogenicity assessment and safety analysis. Therefore, a comprehensive understanding of structure–activity relationships, manufacturing processes, and product quality attributes is essential for successful regulatory approval [189,191].

Future advances in peptide manufacturing are expected to improve the scalability, sustainability, and accessibility of peptide therapeutics. Automation of peptide synthesis, improved flow-based manufacturing approaches, enhanced purification technologies, and computationally guided peptide design may collectively reduce production costs and accelerate development timelines. Moreover, integrating discovery platforms such as phage display with predictive computational approaches may enable the early identification of peptide sequences that not only possess desirable biological functions but also favorable developability profiles. This integration could help bridge the historical gap between peptide discovery and industrial implementation by incorporating manufacturing considerations earlier in the discovery pipeline. Taken together, while phage display has proven highly effective for discovering peptide ligands against diverse biological targets, the journey from a selected peptide sequence to a clinically viable therapeutic requires addressing multiple chemical, technological, and industrial challenges. The future success of phage display-derived peptide therapeutics will therefore depend not only on advances in library design, selection technologies, and computational analysis but also on parallel improvements in peptide engineering, scalable synthesis, purification, and manufacturing. Recognizing these translational challenges is essential for developing peptide therapeutics that are not only biologically promising but also economically feasible, environmentally sustainable, and compatible with modern pharmaceutical production standards. Future efforts in peptide drug discovery will increasingly rely on the integration of molecular design, developability assessment, process development, and manufacturing considerations from the earliest stages of candidate identification. Rather than treating peptide discovery and industrial production as sequential and largely independent processes, future development strategies are expected to adopt a more integrated framework in which peptide engineering, manufacturability, sustainability, and regulatory requirements are considered throughout the entire development process. Such a holistic approach has the potential to improve development efficiency, reduce late-stage attrition, and ultimately accelerate the translation of phage display-derived peptide candidates into safe, effective, and commercially viable therapeutics.

## 6. Clinically Approved Phage Display-Derived Peptides and Disease Treatment: Broadening the Scope of Drug Discovery

Phage display is a promising platform for the development of peptide drugs. Since the birth of phage display four decades ago, many peptides that bind to a wide variety of clinically relevant targets have been isolated from phage display libraries. Many of these peptides have passed successfully through preclinical studies, reaching the different phases of clinical evaluations. Until now, several peptides derived from phage display have received FDA approval for routine clinical applications. The development of these therapeutic peptides positions phage display among the well-established technologies in the field of drug discovery. Here, phage display-derived peptides that have entered the drug market and are prescribed for different diseases are reviewed. For all these peptides, the journey from initial hit identification from phage display libraries to clinical approval has taken many years. Table 3 contains a summarized version of the key information about these peptides.

### 6.1. Romiplostim

Romiplostim, also known as Nplate or AMG 531 and developed by Amgen, is a peptibody composed of a peptide sequence and an Fc domain of IgG1. The peptide component of the drug binds specifically to and activates the thrombopoietin (TPO) receptor. By acting as a highly potent functional mimetic of TPO, a hormone that regulates platelet production, this drug elevates platelet counts in patients with ITP and hematopoietic syndrome of acute radiation syndrome (ARS). ITP is an autoimmune condition characterized by enhanced platelet destruction and impaired platelet production, and the hematopoietic syndrome of ARS is a disease caused by exposure to high amounts of ionizing radiation marked by a reduced number of all blood cell types, including platelets. Romiplostim is also under clinical investigation for other thrombocytopenic conditions. The drug received FDA approval in 2008 for the treatment of chronic ITP, recognized as the first FDA-approved peptibody, and in 2021 for the treatment of hematopoietic syndrome of ARS [194,195].

The TPO receptor-binding peptide sequence in romiplostim was originally discovered by phage display from libraries in which peptides were fused to the pVIII coat protein of the M13 filamentous phage. This peptide sequence later underwent substantial modifications [33]. To develop romiplostim, two identical subunits were formed, each containing two copies of the peptide sequence covalently linked to an Fc fragment of human IgG1. The Fc fragment extends the in vivo half-life of the final product and facilitates its purification during manufacturing. Romiplostim shares no significant sequence homology with TPO, and as a result, it induces a low incidence of anti-drug antibodies that would not be expected to show cross-reactivity with endogenous TPO [196]. This lack of cross-reactivity is clinically significant, as the first-generation recombinant TPO products were discontinued after patients developed antibodies that targeted their own native TPO, causing severe thrombocytopenia and a reliance on platelet transfusion.

### 6.2. Ecallantide

Ecallantide, also known as Kalbitor or DX-88 and developed by Dyax, was discovered by phage display from a library constructed based on the first Kunitz domain (a conserved protease inhibitor domain) of human lipoprotein-associated coagulation inhibitor (LACI-D1) [197]. This drug is a potent, selective, and reversible inhibitor of human plasma kallikrein and was approved in 2009 for the treatment of acute hereditary angioedema (HAE), a rare autosomal dominant disorder caused by a deficiency or dysfunction in C1 esterase inhibitor (C1-INH). C1-INH plays an essential role as an endogenous regulator of plasma kallikrein. In type 1 HAE, patients have low levels of C1-INH, while in type II HAE, the inhibitor is present but becomes nonfunctional. The reduced level of functional activity of C1-INH results in elevated plasma kallikrein activity. During an HAE attack, this uncontrolled activation of plasma kallikrein gives rise to the overproduction of bradykinin, which enhances vascular permeability and causes edema and pain in various parts of the body. By inhibiting kallikrein, ecallantide prevents the generation of bradykinin and inhibits bradykinin-mediated clinical manifestations [198,199].

Ecallantide is composed of 60 amino acids. While peptides often lack stable tertiary structures, the Kunitz domain scaffold used to develop ecallantide is a compact and stable domain with a well-defined tertiary structure containing β-sheet and stabilized by disulfide bonds. Ecallantide was derived from a non-antibody phage display library for engineering of a naturally occurring protease domain [197]. Therefore, the structure of ecallantide makes it closer to a miniprotein than a simple peptide. However, due to its small size, it is described as a peptide mimetic or structured peptide domain. The development of ecallantide highlights the application of phage display in discovering therapeutics derived from novel protein scaffolds, offering an alternative to the more common exploitation of this technology in generating therapeutic antibodies.

### 6.3. Pegcetacoplan

Pegcetacoplan, also known as Empaveli or Syfovre and developed by Apellis Pharmaceuticals, is a PEGylated cyclic peptide that binds to and acts as a potent inhibitor of complement C3, preventing the formation of downstream complement activation products (such as C3a and C5a) that are involved in inflammation and immune response. The original peptide was discovered from a phage display library [200], which was later modified through chemical modification and structural redesign to improve its pharmacological properties and further optimized through PEGylation to increase its stability and prolong its half-life in the circulation. Pegcetacoplan is primarily used for the treatment of diseases pertinent to excessive complement activation, including PNH, which is a rare blood disorder characterized by the complement-associated lysis of defective red blood cells, and GA, which is an age-related macular degeneration (AMD) condition leading to vision loss. Pegcetacoplan received approval in 2021 for the treatment of PNH (under the brand name Empaveli) and in 2023 for the treatment of GA (under the brand name Syfovre) [174,201]. In addition, this drug is under clinical trials for several other complement-mediated diseases, including hematologic, renal, and neurologic disorders. The complement system, in particular C3, is a pivotal and highly conserved component of innate immunity. Due to the need for selective inhibition while avoiding global immune suppression and the existence of complex regulatory feedback loops, targeting the complement system is extremely challenging. The development of pegcetacoplan demonstrates that small peptides discovered by phage display can yield potent, selective, and high-affinity ligands capable of targeting and modulating complex systems.

### 6.4. Purification Peptide Tn 8.2

Xyntha is a recombinant clotting factor VIII (rFVIII) that received FDA approval in 2008 to treat hemophilia A, a genetic disorder caused by deficiency or absence of factor VIII [202]. Xyntha is a genetically engineered variant of the naturally occurring factor VIII, which was developed to supplement the deficient clotting factor and thus prevent bleeding episodes in hemophilia A patients. This drug is a recombinant protein produced in Chinese hamster ovary (CHO) cells and purified through a process in which an affinity chromatography step is performed by using an FVIII-specific peptide. This FVIII-specific ligand, called the Tn 8.2 peptide, was developed by Wyeth Pharmaceuticals (now part of Pfizer) and identified from a phage display library composed of peptides constrained in a disulfide-bridged loop [203]. This affinity ligand is capable of capturing the recombinant factor VIII from expression medium under mild conditions, effectively isolating the recombinant factor from other proteins and contaminants in the production system. This peptide played a critical role in establishing a GMP-compliant affinity purification protocol for large-scale production of Xyntha at Wyeth/Pfizer [204]. The substitution of the antibody-based affinity column with an affinity column based on a synthetic peptide removes the use of human- or animal-derived affinity ligands during purification. This technical advancement represents a major innovation in biopharmaceutical production and addresses some critical safety concerns in the commercial production of recombinant factor VIII, particularly given the historical hesitancy around using plasma-derived proteins in hemophilia therapies. The purification of the recombinant protein drug with the Tn 8.2 peptide makes the final product safer for diseased individuals, in particular for diseases like hemophilia, in which patients need lifelong treatments. Using Tn 8.2 as part of the commercial production pipeline of Xyntha is an example of how phage display technology can be used not only for the discovery of therapeutic peptides but also as a platform for the purification and manufacturing of therapeutic biologics.

### 6.5. Albiglutide

Albiglutide, also known as Tanzeum and developed by GlaxoSmithKline (GSK), is an agonist of the GLP-1 receptor, which received FDA approval in 2014 [205]. It is used as a subcutaneously injectable drug for the treatment of type 2 diabetes alone (in case of inefficacy or intolerance to metformin) or in combination with other antidiabetic drugs. Albiglutide mimics the function of GLP-1, a hormone that increases insulin secretion, leading to reduced glucagon secretion, slower gastric emptying, and finally improved blood glucose control [206]. To develop this drug, phage display was used to identify and optimize GLP-1 variants with improved pharmacological properties, including resistance to dipeptidyl peptidase-4 (DPP-4), which normally breaks down native GLP-1. After selecting the optimal GLP-1 variant, two copies of the modified human GLP-1 analog were fused to human albumin to extend their half-life [206]. This modification allowed for once-weekly dosing [207,208]. In 2017, GSK announced plans to voluntarily withdraw albiglutide from the market by 2018 due to market competition and low commercial demand (outcompeted by other GLP-1 agonists like liraglutide and semaglutide), not safety concerns.

### 6.6. Peginesatide

Peginesatide, also known as Hematide and developed by Affymax in collaboration with Takeda Pharmaceutical, was developed from a peptide originally discovered by phage display [209]. It binds to and activates the erythropoietin receptor (EPOR), leading to erythropoiesis stimulation. Structurally, this erythropoietin-mimicking peptide is composed of two identical 21-residue peptide chains (a dimeric peptide) covalently connected via a PEG linker to improve its pharmacological properties, including solubility, stability, and in vivo half-life. Each peptide chain possesses a single disulfide bond [210]. Peginesatide was administered intravenously or subcutaneously, typically once a month, due to its long half-life, which was a significant advantage over traditional erythropoiesis-stimulating agents. It was structurally unrelated to erythropoietin (EPO); however, it showed a similar receptor activation profile.

Peginesatide received FDA approval in 2012 for the treatment of anemia caused by chronic kidney disease (CKD) in adult patients on dialysis [211]. However, just one year after its FDA approval, peginesatide was recalled and withdrawn from the market due to post-marketing reports of severe anaphylactic reactions, including fatal hypersensitivity in some dialysis patients after the first dose [212]. Despite its withdrawal, peginesatide represents a prominent example of how a synthetic peptide mimetic can be designed to activate a complex receptor and serve as a therapeutic agent. In addition, it shows the prospects and challenges of peptide engineering for receptor targeting and serving as an alternative to protein-based biologics in drug development.

## 7. Concluding Remarks and Future Perspectives

The evolution of phage display technology over the past four decades has fundamentally reshaped the landscape of peptide drug discovery. As we have seen throughout this review, phage display provides a powerful platform for navigating the vast combinatorial diversity of peptide libraries, enabling the identification of high-affinity and high-specificity ligands for an impressive range of biologically relevant targets. The “best-of-both-worlds” features of peptides, blending the advantages of small molecules and antibodies, make them uniquely suited to tackle therapeutic challenges that have eluded traditional drug modalities [115]. However, although the identification of potent binders through phage display has been a remarkable success, translating these hits into clinically viable drugs has necessitated overcoming significant pharmacological limitations. Strategies that are utilized to modify peptides aim to address these limitations and improve the overall drug-like properties of peptides. Technological improvements in peptide modification strategies have enabled tailoring of peptide structure, stability, and bioavailability, addressing major hurdles such as rapid degradation, poor membrane permeability, and short half-life. By applying these innovative modification technologies, researchers can effectively bridge the gap between discovery and therapeutic application, ensuring that phage display-derived peptides realize their full potential as powerful, next-generation medicines. In total, these advances have proven indispensable for transforming initial hits from phage display selections into clinically approved peptide therapeutics that meet urgent medical needs across various disease domains.

The trajectory of phage display in peptide drug discovery underscores its continued promise as a cornerstone technology. As the field moves forward, several emerging technologies and scientific innovations are poised to expand the horizons of what phage display can achieve. One of the most influential developments in recent years has been the integration of NGS into the biopanning workflow [80,213]. Traditional biopanning approaches rely on Sanger sequencing to identify a small subset of enriched clones after selection. While effective, this approach offers only a limited snapshot of the enrichment landscape and often overlooks lower-abundance peptides with therapeutic potential. NGS has revolutionized this process by enabling comprehensive, high-throughput analysis of entire phage pools, providing deep insights into peptide enrichment dynamics, sequence diversity, and evolutionary trajectories during selection rounds [214,215,216]. By utilizing NGS data, researchers can better understand the complex interplay between peptide sequence space and target binding, uncovering not only the most abundant peptides but also less abundant sequences with unique binding profiles that might have otherwise remained undiscovered. This deeper resolution of selection dynamics has already led to the identification of novel peptides with superior properties, and it holds great potential for accelerating the discovery of future therapeutic leads. An additional critical contribution of NGS in phage display is its capacity to reveal and quantify biases introduced during the amplification steps of biopanning [46,51]. Amplification-associated bias can skew the enrichment of certain peptide sequences, favoring those that replicate more efficiently rather than those with the highest target affinity [45,217]. NGS empowers researchers to distinguish true binders from amplification artifacts [218]. By identifying and correcting for these biases, NGS ensures a more accurate interpretation of selection outcomes, ultimately improving the reliability and efficiency of peptide discovery through phage display [46]. In parallel, the emergence of artificial intelligence (AI) is catalyzing a new wave of innovation in phage display-based peptide discovery. AI is uniquely suited to handle the massive and complex datasets generated by NGS, enabling predictive modeling and rational peptide design in ways that were previously unattainable. AI algorithms can identify patterns and features in peptide sequences that correlate with high-affinity binding, stability, and other desirable pharmacological properties. By training on existing datasets of peptide sequences and their corresponding binding data, these models can identify sequence features associated with target binding and predict promising peptide candidates, thereby streamlining the pipeline of ligand discovery. An illustrative example of this approach is CD47Binder, which integrated NGS-derived phage display data with machine-learning techniques to identify CD47-binding peptides [219]. The study evaluated ten traditional machine-learning methods based on multiple peptide descriptors together with three deep learning methods to construct computational models for peptide identification. An integrated predictor based on a support vector machine (SVM), capable of distinguishing target-binding peptides from non-binding peptides, was subsequently developed and implemented as a publicly available web server. This work demonstrates how AI-driven analysis of NGS-derived phage display datasets can accelerate peptide identification and enhance the efficiency of discovering promising peptide ligands. Moreover, AI can be harnessed to guide the design of phage display libraries themselves. Rather than relying solely on random or semi-random peptide sequences, researchers can use AI to generate biased libraries enriched for motifs or sequence features predicted to yield high-affinity binders. As a practical demonstration of this concept, deep sequencing combined with machine learning has been used to extract informative sequence patterns from weakly enriched phage display libraries, in which conventional selection approaches failed to identify functional variants [220]. By analyzing sequence data from appropriate stages of the selection process and using machine learning-based clustering, prospective sequence features associated with functional variants were identified. These features were subsequently incorporated into the design of refined phage display libraries, leading to the discovery of improved target-binding variants. This rational design approach promises to increase the overall efficiency of biopanning experiments, reduce the number of required selection rounds, and ultimately shorten the timeline from initial hit discovery to clinical translation. Outside the context of phage display, AI is also transforming peptide drug discovery through the de novo design and optimization of therapeutic peptides [221,222,223,224,225,226]. By integrating sequence, structural, and functional information, AI-driven models can facilitate the identification of peptides with improved pharmacological properties. Recent advances in generative AI and large language models are further enhancing the ability to design novel peptide sequences with desired biological functions, opening new avenues for the rational development of peptide-based therapeutics. Together, these advances are expected to establish AI as a powerful driver of next-generation peptide engineering, advancing the development of peptide therapeutics with enhanced efficacy, affinity, stability, specificity, and developability.

Rather than considering phage display as an isolated screening technology, its future development may be envisioned as an integrated discovery ecosystem in which optimized library architectures, high-throughput sequencing, computational intelligence, and advanced peptide engineering operate synergistically. In this emerging framework, phage display is expected to evolve beyond a largely empirical selection approach toward a more data-driven and rationally guided platform for exploring peptide sequence space. The combination of experimentally generated diversity with NGS-based quantitative analysis and AI-assisted prediction of peptide properties (e.g., target-binding affinity), together with AI-guided library design, has the potential to transform the way peptide libraries are designed, library biases are detected, selections are interpreted, nonspecific binders are distinguished from target-specific peptides, and target-specific candidate sequences are identified. Such integration may ultimately enable a more knowledge-driven discovery cycle, in which experimental results inform computational models and computational predictions guide the design of improved experimental strategies. This integrated framework could substantially enhance the efficiency, predictability, and translational success of phage display-derived peptide therapeutics.

As we move forward, the continued expansion of the peptide drug market and the growing recognition of the unique advantages of peptides over small molecules and antibodies will contribute to solidifying the role of phage display in the pharmaceutical industry. As new therapeutic targets emerge, particularly those deemed undruggable by conventional small molecule approaches, phage display stands ready to deliver peptide-based solutions that can modulate these targets with high specificity and minimal off-target effects. Equally important is the growing interest in personalized and precision medicine. The ability to rapidly generate target-specific peptides through phage display, combined with AI-driven customization and NGS-informed therapeutic decision-making, could enable the development of individualized peptide therapeutics tailored to specific disease profiles or patient subgroups.

In conclusion, phage display has already established itself as a groundbreaking technology in the discovery of peptide drugs. The field has evolved from initial library selection efforts to the development of clinically approved peptide therapeutics that address significant unmet medical needs. As emerging technologies such as NGS and AI continue to evolve and work in tandem with phage display, the future of peptide drug discovery promises to be even more dynamic and fruitful. The ability to harness these cutting-edge approaches will empower researchers and pharmaceutical innovators to realize the full therapeutic potential of phage display in peptide discovery, propelling this technology into the next chapter of its storied journey and strengthening its place at the forefront of modern drug discovery.

## Figures and Tables

**Table 1:** A comparison of the various biological properties of small molecules, peptides, and antibodies. By occupying the chemical space between small molecules and antibodies, peptides represent a distinct class of drugs that represent many advantages of both systems.

Feature	Small Molecules	Peptides	Antibodies
Molecular weight	<1 kDa	1–5 kDa	>12 kDa
Target specificity	Moderate	High	Very high
Target accessibility	Intracellular and extracellular	Mainly extracellular (can be engineered for intracellular transport; e.g., cell-penetrating peptides or CPPs)	Mostly extracellular (can be engineered to function intracellularly in particular as antibody fragments such as scFv and nanobody)
Cell and tissue permeability	High	Moderate (can be improved with CPPs)	Low
Oral bioavailability	High	Low	Very low
Stability	High	Low to moderate (enhanced via modifications)	High
Plasma half-life	Short	Short (extendable via PEGylation, cyclization, etc.)	Long
Immunogenicity	Low	Low	High
Production cost	Low	Moderate	High
Manufacturing complexity	Low	Moderate	High
Scalability	Very high	High	Moderate
Structural flexibility	High	High	Low
Ease of chemical modification	High	High	Low
Binding selectivity	Low	Low to moderate (when optimized)	Very high
Binding affinity (Kd)	Low	Low to moderate (when optimized)	Very high
Patenting and IP flexibility/novel chemical space	Crowded and mature/limited novelty space	Emerging, growing, and flexible/novel sequences and still patentable platforms	Highly saturated and limited (dominated by platform technologies and large players)/competitive environment
Regulatory pathway maturity	Very mature	Emerging and expanding	Well established
Time to market	Fast	Intermediate	Slow
Mechanism of action versatility	Broad (agonists, antagonists, modulators, etc.)	Broad (receptor mimics, enzyme inhibitors, receptor agonists, etc.)	More limited; mostly neutralization/blockade

**Table 2:** Overview of peptide modification strategies, their advantages, limitations, and resulting improvements in the pharmacological properties of peptides.

Modification Strategy	Advantages	Limitations	Pharmacological Improvement
**Cyclization**	Enables stabilization of secondary structure elements Stabilizes bioactive conformation Expands chemical space Is particularly effective for targeting PPIs Enables the generation of bicyclic peptides with enhanced structural rigidity	Chemical instability of disulfide-based cyclization Reduced library size in chemical synthesis approaches Limited scaffold diversity in some cyclization strategies Challenging optimization of ring size and structural diversity	Increased binding affinity, selectivity, protease resistance, structural stability, membrane permeability (particularly backbone cyclization) Reduced entropic penalty upon target binding Improved retention of affinity after removal from the phage scaffold
**Incorporation of ncAAs**	Expands chemical and functional diversity Enables novel functionalities and rational peptide engineering Enables genetic code expansion in phage display	Low efficiency and fidelity of incorporation Limited codon availability Reduced phage yield and fitness Toxicity or poor permeability of some ncAAs Reduced library complexity Less flexibility of phage display compared to cell-free systems	Increased chemical diversity, structural diversity, and proteolytic stability Introduction of new functionalities (reactive groups, cyclization handles, β-amino acids, etc.)
**Introduction of D-amino acids**	Provides excellent resistance to proteolysis Provides access to highly stable peptide therapeutics Is generally less immunogenic Allows identification by mirror image phage display	Required synthesis of D-protein targets Mainly applicable to peptides and small proteins as the target	Increased serum stability, resistance to proteolytic degradation, circulation time, and bioavailability Potentially reduced immunogenicity
**Oligomerization**	Is particularly useful for cell surface targets with high receptor density Preserves activity after removing peptides from the phage scaffold Provides flexible architectures (e.g., dimers, trimers, tetramers, branched structures) Mimics multivalent phage display Provides capacity for affinity maturation	Increased molecular complexity Dependence of its effectiveness on receptor density and spatial organization	Increased apparent affinity (avidity), cellular targeting, biological activity, serum stability, receptor engagement, and efficiency of targeted delivery
**Conjugation/Fusion**	Improves the pharmacokinetic profile Uses biologically compatible carriers Provides a clinically validated strategy for peptide therapeutics Provides the ability to perform site-specific conjugation Facilitates interaction with cell membranes (in the case of lipidation)	Heterogeneous conjugation products Increased manufacturing complexity Reduced peptide folding or activity due to fusion partners	Increased plasma half-life and circulation time Prolonged duration of action Reduced renal clearance Improved therapeutic efficacy
**Stapling**	Is particularly effective for modulating PPIs mediated by α-helices Preserves biologically active conformations Enables the development of constrained peptide modulators Stabilizes α-helices	Manufacturing complexity and cost Potential immunogenicity Significant delivery challenges Required optimization of staple position and chemistry	Increased helical stability, binding affinity, proteolytic stability, cell permeability, and structural rigidity

**Table 3:** Clinically approved peptide drugs developed by phage display technology.

	Romiplostim	Ecallantide	Pegcetacoplan	Tn 8.2	Albiglutide	Peginesatide
**Alternative Names**	Nplate, AMG 531	Kalbitor, DX-88	Empaveli, Syfovre	Tn 8.2 purification peptide	Tanzeum	Hematide
**Target**	TPOR	Plasma kallikrein	C3 protein	rFVIII	GLP-1 receptor	EPOR
**Mechanism of Action**	Agonism	Antagonism	Antagonism	Affinity capture of recombinant factor VIII from expression medium	Agonism	Agonism
**Developer**	Amgen	Dyax	Apellis Pharmaceuticals	Wyeth/Pfizer	GSK	Affymax/Takeda Pharmaceutical
**Application**	Treatment of ITP and hematopoietic ARS	Treatment of HAE	Treatment of PNH and GA	Purification of Xyntha	Treatment of type 2 diabetes	Treatment of anemia in CKD patients
**Year of Market Entry**	2008 for ITP 2021 for hematopoietic ARS	2009 for HAE	2021 for PNH 2023 for GA	-	2014	2012
**Notes**	Peptibody with peptide sequences attached to an IgG1 Fc fragment	Developed based on the first Kunitz domain of human LACI-D1	Cyclic, attached to PEG	Cyclic, an affinity ligand (not a peptide drug) used in the commercial purification of the FDA-approved drug Xyntha	Attached to human albumin, withdrawn from the market due to commercial reasons	Attached to PEG, withdrawn from the market due to safety concerns

## Data Availability

No new data were created or analyzed in this study. Data sharing is not applicable to this article.

## Abbreviations

ACE2	Angiotensin-converting enzyme 2
AFPS	Automated fast-flow peptide synthesis
AI	Artificial intelligence
ALS	Amyotrophic lateral sclerosis
AMD	Age-related macular degeneration
Anti-AChR	Anti-acetylcholine receptor
APCs	Antigen-presenting cells
APIs	Active pharmaceutical ingredients
ARS	Acute radiation syndrome
C1-INH	C1 esterase inhibitor
C5	Complement component 5
CAGR	Compound annual growth rate
CD44	Cluster of differentiation 44
CHIP	C-terminus of Hsc70-interacting protein
CHO	Chinese hamster ovary
CKD	Chronic kidney disease
CPPs	Cell-penetrating peptides
CXCL8	C–X–C motif chemokine ligand 8
DECLs	DNA-encoded chemical libraries
DPP-4	Dipeptidyl peptidase-4
EGFR	Epidermal growth factor receptor
EPOR	Erythropoietin receptor
FACS	Fluorescence-activated cell sorting
FGFR	Fibroblast growth factor receptor
FIT	Flexible in vitro translation
FXIa	Activated coagulation factor XIa
GA	Geographic atrophy
GABARAP	γ-Aminobutyric acid receptor-associated protein
GAGs	Glycosaminoglycans
GCE	Genetic code expansion
GLP-1	Glucagon-like peptide-1
gMG	Generalized myasthenia gravis
GMP	Good manufacturing practice
GSK	GlaxoSmithKline
HAE	Hereditary angioedema
HDM2	Human double minute 2
HER2	Human epidermal growth factor receptor 2
hKeap1	Human Kelch-like ECH-associated protein 1
HTS	High-throughput screening
IMNM	Immune-mediated necrotizing myopathy
ITP	Immune thrombocytopenic purpura
IVT	In vitro translation
LACI-D1	First Kunitz domain of human lipoprotein-associated coagulation inhibitor
LDLR	Low-density lipoprotein receptor
LN	Lupus nephritis
LTS	Low-throughput sequencing
MDM2	Mouse double minute 2
NEK7	NIMA-related kinase 7
NGS	Next-generation sequencing
ncAAs	Non-canonical amino acids
OBOC	One-bead-one-compound
o-NBAK	o-nitrobenzyl alcohol lysine
OPA	ortho-phthalaldehyde
PCSK9	Proprotein convertase subtilisin/kexin type 9
PEG	Polyethylene glycol
PNH	Paroxysmal nocturnal hemoglobinuria
PPIs	Protein–protein interactions
PSA	Prostate-specific antigen
PTP1B	Protein tyrosine phosphatase 1B
PURE	Protein synthesis using recombinant elements
RaPID	Random non-standard peptide integrated discovery
RBD	Receptor-binding domain
R&D	Research and development
rFVIII	Recombinant factor VIII
RI	Retro-inverso
SARS-CoV-2	Severe acute respiratory syndrome coronavirus 2
SICLOPPS	Split-intein circular ligation of proteins and peptides
siRNA	Small interfering RNA
SPSS	Solid-phase peptide synthesis
SVM	Support vector machine
TBI	Traumatic brain injury
TBMB	tris-(bromomethyl)benzene
TPO	Thrombopoietin
UAG	Amber stop codon
uPA	Urokinase-type plasminogen activator
VEGF	Vascular endothelial growth factor

## Appendix A

The following selection of passages from Umberto Eco’s *The Name of the Rose* [227] is included as a literary and philosophical reflection on the conceptual themes underlying this review. In Eco’s novel, the library is simultaneously a repository of books, a labyrinth of knowledge, and a space in which signs must be interpreted to extract meaning. This imagery closely connects with the central themes of the present review, in which phage display libraries similarly constitute vast spaces of molecular information, with individual peptide sequences corresponding analogously to the books contained within the library. The search for meaning or truth within Eco’s library thus finds a parallel in the search for biologically meaningful sequences within peptide libraries. In this context, the reading of books can also be viewed metaphorically as the sequencing of the library, with deep sequencing providing a means of reading and interpreting its vast molecular information.

The particular selection and juxtaposition of passages presented here was inspired by a compilation of quotations from the novel by Richard Smith in “*Umberto Eco on Books*”. The passages were independently verified against the English translation by William Weaver.

*The library is a great labyrinth, sign of the labyrinth of the world. You enter and you do not know whether you will come out.*
*.....And the library was built by a human mind that thought in a mathematical fashion, because without mathematics you cannot build labyrinths.*
*.....The library is testimony to truth and to error.*
*.....We live for books [of the library]; a sweet mission in this world dominated by disorder and decay.*
*.....Here we are trying to understand what has happened among men who live among books, with books, from books, and so their words on books are also important.*
*.....The good of a book lies in its being read. A book is made up of signs that speak of other signs, which in their turn speak of things. Without an eye to read them, a book contains signs that produce no concepts; therefore it is dumb.*

*The Name of the Rose*
*Umberto Eco*
*(Translated by William Weaver)*
